# Direct sulfuric acid formation from the gas-phase oxidation of reduced-sulfur compounds

**DOI:** 10.1038/s41467-023-40586-2

**Published:** 2023-08-10

**Authors:** Torsten Berndt, Erik H. Hoffmann, Andreas Tilgner, Frank Stratmann, Hartmut Herrmann

**Affiliations:** 1 Atmospheric Chemistry Department (ACD), Leibniz Institute for Tropospheric Research (TROPOS), 04318 Leipzig, Germany; 2 Atmospheric Microphysics Department (AMP), Leibniz Institute for Tropospheric Research (TROPOS), 04318 Leipzig, Germany

**Keywords:** Atmospheric chemistry, Atmospheric chemistry

## Abstract

Sulfuric acid represents a fundamental precursor for new nanometre-sized atmospheric aerosol particles. These particles, after subsequent growth, may influence Earth´s radiative forcing directly, or indirectly through affecting the microphysical and radiative properties of clouds. Currently considered formation routes yielding sulfuric acid in the atmosphere are the gas-phase oxidation of SO_2_ initiated by OH radicals and by Criegee intermediates, the latter being of little relevance. Here we report the observation of immediate sulfuric acid production from the OH reaction of emitted organic reduced-sulfur compounds, which was speculated about in the literature for decades. Key intermediates are the methylsulfonyl radical, CH_3_SO_2_, and, even more interestingly, its corresponding peroxy compound, CH_3_SO_2_OO. Results of modelling for pristine marine conditions show that oxidation of reduced-sulfur compounds could be responsible for up to ∼50% of formed gas-phase sulfuric acid in these areas. Our findings provide a more complete understanding of the atmospheric reduced-sulfur oxidation.

## Abstract (web summary)

Experiments verify that oxidation of organic sulphur compounds, such as DMS, directly yields gas-phase H_2_SO_4_. Simulations reveal that this pathway can be competitive with SO_2_ oxidation over oceans in the Southern Hemisphere.

## Introduction

Since more than 3 decades, reduced organic sulfur compounds have been recognized as substantial biogenic emissions contributing to Earth´s sulfur cycle. The sulfur cycle is highly relevant for the Earth’s climate due to the ability of the sulfur compound’s oxidation products, sulfuric acid (H_2_SO_4_) and methane sulfonic acid (MSA, CH_3_SO_3_H), to generate new airborne particles that effectively scatter incoming solar radiation and affect the formation of cloud condensation nuclei (CCN)^1,2^. CCN in turn may have significant influences on the microphysical and radiative properties^3^ and lifetime^4^ of clouds.

**Fig. 1:**
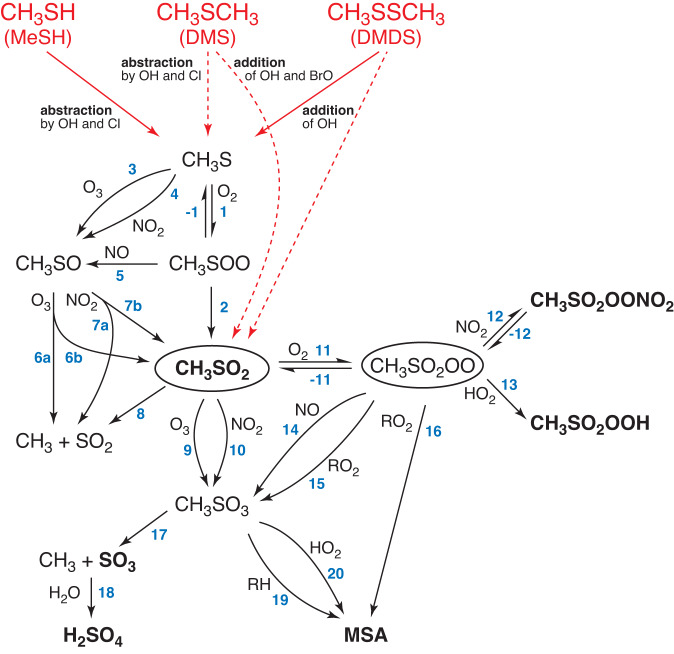
Reaction scheme of the oxidation of reduced-sulfur emissions, i.e. CH_3_SH (MeSH, methylthiol), CH_3_SCH_3_ (DMS, dimethyl sulfide) and CH_3_SSCH_3_ (DMDS, dimethyl disulfide). The scheme focuses on the reaction steps relevant for the formation of H_2_SO_4_ and MSA (methane sulfonic acid) starting from CH_3_S and CH_3_SO_2_ and is mainly based on Barnes et al.^9^. Signals of observed products in the present study are shown in bold. Dashed red arrows indicate complex reactions to the stated intermediates. Only important main products of the individual pathways are displayed.

Globally, the most important organic sulfur compound is dimethyl sulfide (DMS, CH_3_SCH_3_) with an annual emission rate of ∼30 million metric tons of sulfur, followed by methylthiol (MeSH, CH_3_SH) and, to a lesser extent, dimethyl disulfide (DMDS, CH_3_SSCH_3_)^5^. A large number of experimental and theoretical studies have been conducted to ascertain their atmospheric degradation pathways, especially for DMS^6–18^, representing the data base for atmospheric models^19–22^. The reaction scheme in Fig. 1 summarises the current knowledge on product formation starting from the methylthiyl (CH_3_S) and methylsulfonyl radical (CH_3_SO_2_), both formed as important intermediates in the gas-phase oxidation of CH_3_SH, DMS and DMDS. To the best of our knowledge, up to now there is no experimental evidence for the direct gas-phase formation of H_2_SO_4_, other than via SO_2_ oxidation by OH radicals^23,24^ or Criegee intermediates^25,26^, although this has been speculated about in the literature for long^27,28^ and such pathways have already been implemented in models^19–22^.

Here we experimentally demonstrate the direct formation of H_2_SO_4_ from the OH radical-initiated gas-phase oxidation of organic sulfur compounds by its direct mass spectrometric detection in two flow systems^29–31^ under atmospheric conditions with residence times of 7.9 and 32 s. Accompanied modelling shows the importance of this direct pathway for the total H_2_SO_4_ formation in the atmosphere.

## Results and discussion

### Detectable products from CH_3_S oxidation

**Fig. 2:**
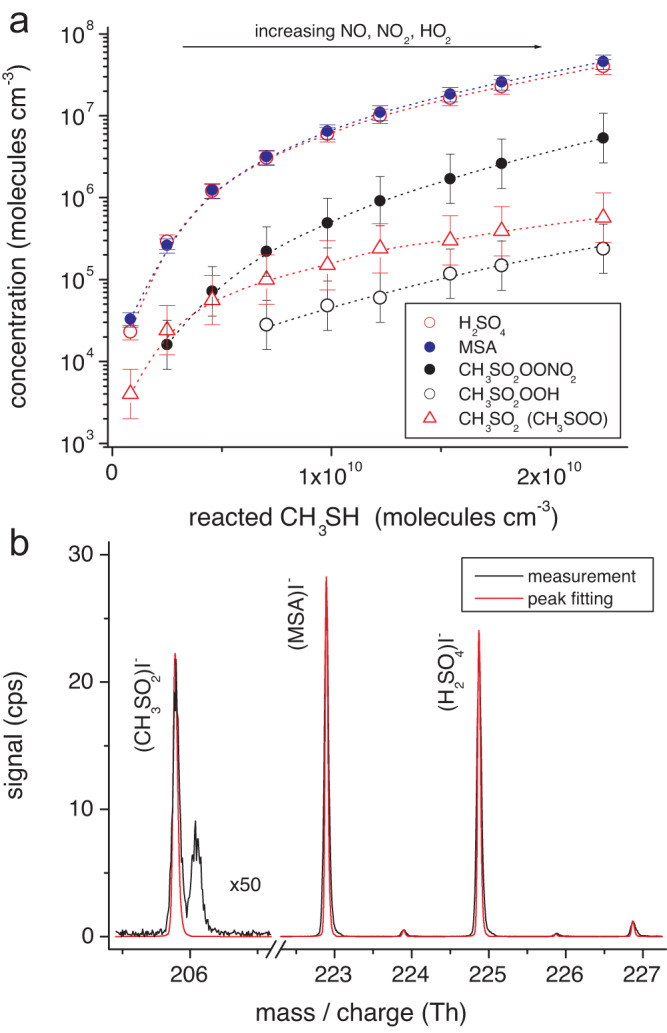
Product detection from CH_3_S oxidation using ionisation by iodide. Experiments on OH + CH_3_SH for CH_3_S production have been conducted in the free-jet flow system, t = 7.9 s, at r.h. = 10%. OH radicals were produced from IPN (isopropyl nitrite) photolysis, i.e. OH radical generation via NO + HO_2_ → NO_2_ + OH. Increasing OH radical levels for rising CH_3_SH conversion were linked by increasing concentrations of NO, NO_2_ and HO_2_ due to increasing IPN conversion in the photolysis. Reactant concentrations are stated in Supplementary Table 1. Source data are provided as a Source Data file. **a** Detected products are given as a function of converted CH_3_SH. Data for H_2_SO_4_ and MSA (methane sulfonic acid) are based on absolute calibration with an uncertainty of ∼20%. Other concentrations represent lower limits with an uncertainty of a factor of 2. **b** Measured raw spectrum from 10 min data accumulation compared with calculated signals of iodide adducts with CH_3_SO_2_, H_2_SO_4_ and MSA from peak fitting.

Product ionization by means of iodide (I^-^) and nitrate (NO_3_^-^) in the mass spectrometric analysis was found as a suitable way to observe product formation, other than SO_2_, in the oxidation process. Recently, an experimental SO_2_ yield of 86 ± 18% has been reported for low-NO conditions qualifying SO_2_ as the predominant product^32,33^. Figure 2a shows the detected products, other than SO_2_, from an overview experiment on the oxidation of CH_3_S initiated by the reaction OH + CH_3_SH → CH_3_S + H_2_O^33,34^ (Fig. 1). Product ionisation by iodide allowed to follow H_2_SO_4_ and MSA, signals consistent with the formation of CH_3_SO_2_OONO_2_ and CH_3_SO_2_OOH, which were most likely formed via pathways 12 and 13 (Fig. 1), respectively, and the signal of the intermediate CH_3_SO_2_. For the latter, a contribution from CH_3_SOO cannot be ruled out. It is to be noted here, that H_2_SO_4_ formation initiated by OH + SO_2_ is unimportant under the chosen conditions and, thus, H_2_SO_4_ needs to arise from the CH_3_S oxidation directly, see also Methods. Comparison of the measured product spectra with results from peak fitting of the spectra supports the signal attribution to the five products identified (Fig. 2b and Supplementary Fig. 1). The signals of all closed-shell products steeply increased with rising CH_3_SH conversion while the CH_3_SO_2_ signal levelled off, typical for reactive intermediates. The occurrence of CH_3_SO_2_OONO_2_ and CH_3_SO_2_OOH, recently detected from OH + DMS as well^14^, indicates CH_3_SO_2_OO as a significant peroxy species in these reaction systems, which is supported by theoretical calculations^35^.

Basically, the direct observation of CH_3_SO_2_ and other intermediates of the CH_3_S oxidation for close to atmospheric conditions appears to be very challenging^36,37^. A spectroscopic study on the product formation of CH_3_S + O_2_ in cryogenic matrixes unambiguously identified CH_3_SOO, CH_3_SO_2_ and CH_3_SO_2_OO as important intermediates supporting the relevance of the reaction sequence 1/−1, 2 and 11/−11 (Fig. 1) in the CH_3_S oxidation^15^. Cryogenic matrix techniques in general represent an useful approach for qualitative studying sulfur oxidation^38^.

Formation of H_2_SO_4_ and MSA in our experiments was also observed by means of nitrate ionisation confirming the findings using iodide ionisation (Supplementary Fig. 2).

### MSA formation induced by elevated CH_3_SH and DMDS concentrations

**Fig. 3:**
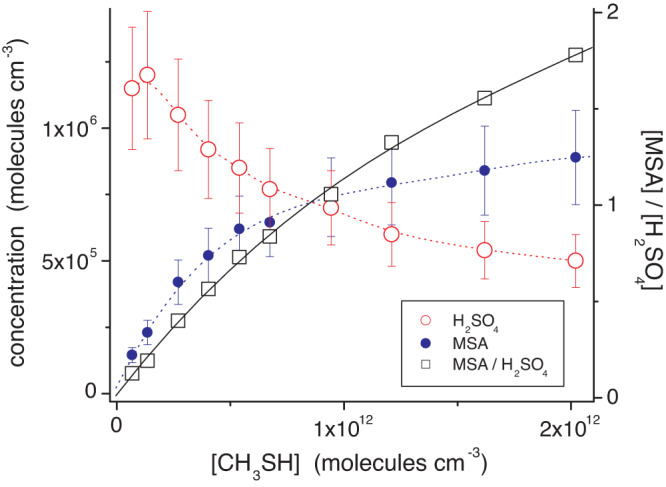
Concentrations of H_2_SO_4_ and MSA (methane sulfonic acid), and the MSA/H_2_SO_4_ ratio as a function of CH_3_SH concentration. Experiments on OH + CH_3_SH were carried out in the free-jet flow system at r.h. = 10% and a reaction time of 7.9 s using IPN (isopropyl nitrite) photolysis for OH radical generation. Reactant concentrations are stated in Supplementary Table 1. The error bars for H_2_SO_4_ and MSA depict the uncertainty of ∼20% based on the uncertainty in the calibration factor. Source data are provided as a Source Data file.

It is remarkable that H_2_SO_4_ and MSA concentrations increased almost uniformly with rising CH_3_SH conversion, which was accompanied by a rising HO_2_ radical level for the chosen reaction conditions (Fig. 2a and Supplementary Fig. 2). The competing steps 17 vs. 20 imply a decreasing H_2_SO_4_/MSA ratio with rising HO_2_ concentrations (Fig. 1), which is not visible in the experiments (Supplementary Fig. 3). This means that our experimental findings do not support considerable MSA formation via CH_3_SO_3_ + HO_2_ (pathway 20). Moreover, an increasing H_2_SO_4_/MSA ratio with decreasing CH_3_SH concentration was observed for otherwise nearly constant reaction conditions, including CH_3_SH consumption by the OH reaction and the prevailing HO_2_ concentration (Fig. 3). The MSA signal practically disappeared for CH_3_SH concentrations below a few 10^10^ molecules cm^−3^. Thus, the reaction of CH_3_SO_3_ with CH_3_SH (pathway 19**)**, likely via H-abstraction of the labile S-bound H atom, seems to dominate the MSA formation under the present conditions. This also means that the direct H_2_SO_4_ formation via CH_3_SO_3_ decomposition (pathway 17**)** is supressed in the presence of sufficiently high CH_3_SH or other substances serving as H-atom donor. A similar behaviour of the H_2_SO_4_/MSA ratio was also observed in OH + DMDS experiments varying the DMDS concentrations (Supplementary Fig. 4). Here, almost exclusive H_2_SO_4_ formation can be expected for DMDS concentrations below 10^10^ molecules cm^−3^.

Because atmospheric CH_3_SH and DMDS concentrations are clearly smaller than 10^10^ molecules cm^−3^ (400 ppt), see attached Supplementary Dataset 1 and ref. ^5^, CH_3_SO_3_ decomposition (pathway 17) forming finally the direct H_2_SO_4_ most likely dominates the fate of CH_3_SO_3_ for atmospheric conditions (Fig. 3 and Supplementary Fig. 4). MSA formation according to CH_3_SO_3_ + RH for RH ≡ CH_3_SH or DMDS (pathway 19) has to be of minor importance. It is speculative whether or not other hydrocarbons RH in the atmosphere could efficiently form MSA via pathway 19.

### Formation routes to direct H_2_SO_4_

**Fig. 4:**
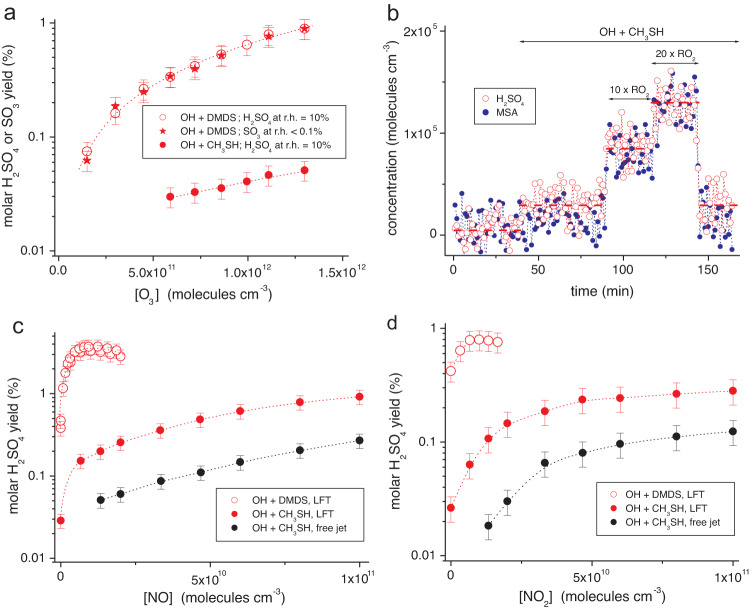
Impact of trace gases on direct H_2_SO_4_ formation using ionisation by nitrate. Experiments were conducted either in the free-jet flow system, t = 7.9 s, or in the laminar flow tube (LFT), t = 32 s, at r.h. = 10% (or <0.1%) using tetramethyl ethylene (TME) ozonolysis for OH radical production. Reactant concentrations are given in Supplementary Table 1. Error bars represent the uncertainty of ∼20% in the absolute calibration. Source data are provided as a Source Data file. **a** Formation of H_2_SO_4_ and SO_3_ as a function of ozone measured in the LFT. Reacted CH_3_SH was in the range (7.6–17) × 10^7^ and (2.1–18) × 10^7^ molecules cm^−3^ for DMDS (dimethyl disulfide). **b** H_2_SO_4_ and MSA (methane sulfonic acid) formation from OH + CH_3_SH depending on RO_2_ radical concentrations, CH_3_C(O)CH_2_O_2_ and CH_3_O_2_, in the LFT. Rising RO_2_ levels were achieved by stepwise increase of TME and corresponding CH_4_ additions keeping reacted CH_3_SH constant at ∼7.6 × 10^7^ molecules cm^−3^. Highest CH_3_C(O)CH_2_O_2_ and CH_3_O_2_ concentrations were 1.0 × 10^9^ and 8.8 × 10^8^ molecules cm^−3^, respectively, calculated from an extended model (Supplementary Table 4). **c** H_2_SO_4_ formation yields as a function of NO addition. **d** H_2_SO_4_ formation yields as a function of NO_2_ addition.

We evaluated the impact of atmospheric trace gases, i.e. ozone, RO_2_ radicals, NO and NO_2_, in the process of direct gas-phase H_2_SO_4_ formation with separate experiments starting from the OH radical reactions with CH_3_SH and DMDS (Fig. 4). While OH + CH_3_SH represents a clean source of CH_3_S with a reported formation yield of 1.1 ± 0.2^34^, OH + DMDS is expected to form CH_3_S and CH_3_SOH^39,40^, which further reacts with ozone leading mainly to CH_3_SO_2_ with a yield close to unity for high enough ozone concentrations^32^. The OH + DMS reaction was not considered in these experiments because of its complexity^9,11^, which complicates the investigation of selected pathways. Reaction conditions were chosen in such a way that intermediate concentrations were kept as low as possible in order to suppress unwanted bimolecular steps not relevant in the atmosphere. For this reason, the amount of reacted CH_3_SH and DMDS was limited to a few 10^8^ molecules cm^−3^. Gas-phase H_2_SO_4_ formation starting from the reaction of SO_2_ with OH radicals or Criegee intermediates was again small in these measurement series and did not influence the results of direct H_2_SO_4_ formation significantly, see also Methods.

Ozone: No significant H_2_SO_4_ formation from OH + CH_3_SH was observed for ozone concentrations of up to 2 × 10^12^ molecules cm^−3^ (∼80 ppb) in the free-jet flow system with the short reaction time of 7.9 s. H_2_SO_4_ became detectable in the laminar flow tube (LFT) with a reaction time of 32 s indicating a relatively slow process of direct H_2_SO_4_ formation (Fig. 4a). Big differences in the H_2_SO_4_ yields of more than an order of magnitude were measured using either OH + CH_3_SH for CH_3_S generation or OH + DMDS forming CH_3_S and most likely CH_3_SO_2_ with high yields. Considering CH_3_SO_2_ as the needed intermediate for direct H_2_SO_4_ formation (Fig. 1), CH_3_S´s oxidation obviously proceeds only with a small share via CH_3_SO_2_, e.g. ≤ 9% for an ozone concentration of 5.7 × 10^11^ molecules cm^−3^ (Fig. 4a) taking OH + DMDS with a CH_3_SO_2_ yield of unity as the reference. Moreover, OH + CH_3_SH experiments with heavy ozone (^18^O_3_) revealed the absence of H_2_SO_4_ containing three ^18^O atoms (Supplementary Fig. 5) as expected from the reaction sequence 3, 6b and 9 (Fig. 1). We largely measured H_2_SO_4_ with one ^18^O atom consistent with the reaction sequence 1/−1, 2 and 9. The findings imply the dominance of pathway 6a over 6b or in fact the irrelevance of pathway 6b, allowing for the importance of ozone reactions in the CH_3_S oxidation^8,34,41^. This can be explained by the high exothermicity of the CH_3_SO + O_3_ reaction forming the chemically excited CH_3_SO_2_* that rapidly decomposes to SO_2_ and CH_3_ before it is thermalised^42^.

The small H_2_SO_4_ yields < 1% for atmospheric ozone concentrations, even under conditions of the preferred CH_3_SO_2_ generation from OH + DMDS, support the efficient decomposition of CH_3_SO_2_ (pathway 8), which is in line with the high SO_2_ yields reported recently^32,33^. SO_3_ yields measured under dry conditions, r.h. < 0.1%, were in very good agreement with the H_2_SO_4_ yields at r.h. = 10% (Fig. 4a) confirming SO_3_ as the precursor of H_2_SO_4_ (pathways 17 and 18 in Fig. 1).

An ozone concentration of 5.7 × 10^11^ molecules cm^−3^ (∼23 ppb) was chosen in the following experiments, which stands for an average concentration over pristine oceans^43^, making our findings applicable to the atmospheric reaction system.

RO_2_ radicals: We detected a distinct impact of RO_2_ radicals on the formation of H_2_SO_4_ and MSA (Fig. 4b). Main RO_2_ radicals in the reaction system are CH_3_C(O)CH_2_O_2_, formed in the course of OH radical generation via TME ozonolysis^44,45^, and CH_3_O_2_ as the by-product of SO_2_ in the oxidation of CH_3_SH and DMDS^32,33^ as well as from OH + CH_4_ in the case of CH_4_ additions. In the OH + CH_3_SH reaction, we increased in a two-step process the concentrations of CH_3_C(O)CH_2_O_2_ and CH_3_O_2_ radicals, first by a factor of ∼10, i.e. from 6.2 × 10^7^ to 5.7 × 10^8^ and from 4.0 × 10^7^ to 4.4 × 10^8^ molecules cm^−3^, respectively, leading to enhanced H_2_SO_4_ formation by a factor of ∼3.5 for constant CH_3_SH conversion (Fig. 4b). Further doubling of the RO_2_ concentrations led to further rise in H_2_SO_4_ productions. The MSA formation, however, increased stronger than that of H_2_SO_4_, which became more visible from a similar experiment on OH + DMDS (Supplementary Fig. 6). Furthermore, we observed predominate MSA formation in a reaction system with HO-C_6_H_12_O_2_ along with CH_3_C(O)CH_2_O_2_ as the main RO_2_ radicals (Supplementary Fig. 7). It can be speculated that most likely CH_3_SO_2_OO reacted with RO_2_ radicals either via the alkoxy channel (pathway 15 in Fig. 1), forming finally H_2_SO_4_, or via the dismutation channel (pathway 16 in Fig. 1), similar to the well-known chemistry of carbon-centred RO_2_ radicals^46^, leading to MSA. The branching ratio of pathways 15 vs. 16 appears to be dependent on the structure of the reacting RO_2_ radical. Other RO_2_ driven pathways, influencing the product formation, cannot be ruled out.

Nitrogen oxide (NO): Addition of NO substantially accelerated the H_2_SO_4_ formation in all experiments (Fig. 4c) supporting the potential importance of CH_3_SO_2_OO for H_2_SO_4_ formation, here via pathway 14 (Fig. 1). An increase in the H_2_SO_4_ production by a factor of ∼4 (Supplementary Fig. 8) was measured using a NO concentration of 1 × 10^9^ molecules cm^−3^ similar to the behaviour observed for elevated RO_2_ concentrations (Fig. 4b and Supplementary Fig. 6). This indicates rate coefficients k_14_ and k_15_ for the reaction of CH_3_SO_2_OO with NO and RO_2_, respectively, being in the same range. Comparison of results for relatively low NO concentrations of < 10^10^ molecules cm^−3^ in the LFT showed more than one order of magnitude higher H_2_SO_4_ yields from the oxidation of DMDS relative to CH_3_SH, in line with the findings from the pure ozone-driven reaction (Fig. 4a). For elevated NO levels, other NO reactions presumably disturbed the CH_3_SO_2_ formation from OH + DMDS and inhibited further rise of the H_2_SO_4_ yield. Further NO reactions in the CH_3_S oxidation could also negatively impact the H_2_SO_4_ formation, such as CH_3_S + NO → CH_3_SNO^10^ or CH_3_SOO + NO → CH_3_SO + NO_2_^10^ (pathway 5) resulting finally in SO_2_ formation via pathway 6a (Fig. 1). The higher H_2_SO_4_ yields from OH + CH_3_SH measured in the LFT at t = 32 s point again to a slow process of H_2_SO_4_ formation that is far away from completeness for the reaction time of 7.9 s in the free-jet flow system.

Nitrogen dioxide (NO_2_): Addition of NO_2_ featured a similar effect for the rise of H_2_SO_4_ yields (Fig. 4d) as observed for NO (Fig. 4c), albeit the NO_2_ impact was less pronounced. It is supposed in the literature that NO_2_ reacts with CH_3_SO_2_ forming CH_3_SO_3_ (pathway 10) which finally leads to H_2_SO_4_ analogous to the ozone-mediated route (pathway 9)^9^. This set of experiments confirmed again the much higher potential of H_2_SO_4_ formation starting from OH + DMDS regarding OH + CH_3_SH, as well as the slow formation rate of the direct H_2_SO_4_ production. H_2_SO_4_ production almost doubled as the result of a NO_2_ addition of 6.7 × 10^9^ molecules cm^−3^ in the LFT experiments (Fig. 4d), indicating nearly the same reaction rate in the reaction of CH_3_SO_2_ with ozone and NO_2_ (pathways 9 and 10 in Fig. 1), [O_3_] = 5.7 × 10^11^ molecules cm^−3^. This leads to *k*_*9*_/*k*_*10*_ ∼ 1/85 being in good agreement with the rate coefficient ratio currently used in models^21,22^. The experiments with NO_2_ addition did not allow any conclusions regarding the relative importance of the product channels 7a and 7b from CH_3_SO + NO_2_.

In summary, the experiments provided evidence for the promoting effect of each of the four important trace gases for the direct H_2_SO_4_ formation. The relatively strong impact caused by RO_2_ radicals and NO (Fig. 4b and 4c) was surprising, which further highlights CH_3_SO_2_OO radicals as important intermediates.

### Application to the atmosphere

Adjustments in the H_2_SO_4_ yields were needed in order to apply the laboratory findings for atmospheric conditions. The incompleteness of the CH_3_SO_3_ conversion due to the short reaction times led to a correcting factor of 1.6 for the H_2_SO_4_ yields in the LFT using *k*_*17*_ = 0.076 s^−1^ at 295 ± 2 K, see Methods. Relatively high CH_3_SH and DMDS concentrations in the experiments, not present in the atmosphere, necessitated further adjustment by a factor of ∼1.5 to allow for the suppression of H_2_SO_4_ formation in their competing reaction with CH_3_SO_3_ forming MSA (pathway 19 in Fig. 1), see Fig. 3 and Supplementary Fig. 4. Adjusted H_2_SO_4_ yields for low-NO_x_ conditions and [O_3_] = 5.7 × 10^11^ molecules cm^−3^ were estimated to be 0.074 ± 0.015% per formed CH_3_S and 0.82 ± 0.02% per formed CH_3_SO_2_ (Fig. 4a) assuming a CH_3_SO_2_ yield of unity from OH + DMDS. The yields increased to 0.11 ± 0.02% (CH_3_S) and 1.2 ± 0.2% (CH_3_SO_2_) incorporating the “RO_2_ effect” (Fig. 4b) for total RO_2_ radical concentrations of ∼3 × 10^8^ molecules cm^−3^, that represents an average RO_2_ level during main CH_3_S and CH_3_SO_2_ production at noon (Supplementary Fig. 9). The ratio *k*_*8*_*/(k*_*9*_ *×* *[O*_*3*_*])* ∼ 120 (*k*_*8*_*/k*_*9*_ ∼ 7 × 10^13^ molecules cm^−3^) followed from the ozone-driven experiments on OH + DMDS with a H_2_SO_4_ yield of 0.82%, that strongly favours SO_2_ formation from CH_3_SO_2_ (pathway 8) being consistent with the high SO_2_ yields measured^32,33^. This *k*_*8*_*/k*_*9*_ ratio, however, is in contrast to the implementation in latest atmospheric models, *k*_*8*_*/k*_*9*_ = 9.5 × 10^11^ molecules cm^−3^ ^21,22^, leading to severe overestimation of the modelled CH_3_SO_3_ production.

### Atmospheric impact

**Fig. 5:**
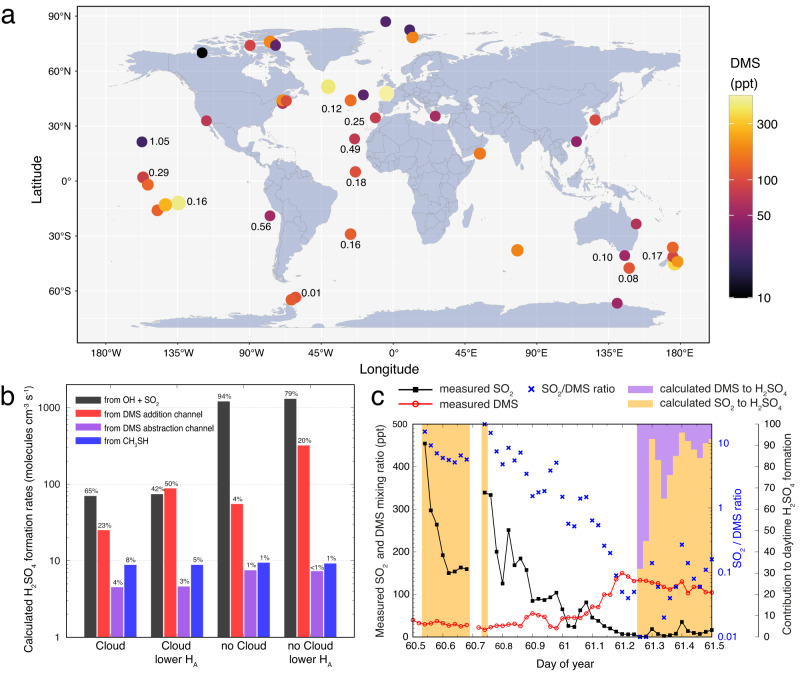
Measured DMS (dimethyl sulfide) and SO_2_ data and the contribution of different pathways to H_2_SO_4_ formation. Source data are provided as a Source Data file. **a** Measured average DMS concentrations (colour coded) worldwide with corresponding SO_2_/DMS ratios (numbers), if available. The map plot was created with R^68^ using the ggplot2 package (map_data(“world”)). **b** Calculated H_2_SO_4_ formation rates from different pathways combining the modelled CH_3_S and CH_3_SO_2_ formation rates with experimentally determined H_2_SO_4_ yields. The “Cloud” scenario represents simulations with cloud passages leading to lower SO_2_ concentrations due to its uptake and oxidation in clouds. The simulations with “lower H_A_” represent model runs using lower Henry constants H_A_ of DMS oxidation products (see Supplementary Table 1). **c** Reproduction of measured SO_2_ (black line with squares) and DMS (red line with dots) concentrations as well as its SO_2_/DMS ratio (blues crosses) from observations at Baring Head, New Zealand^50^. Purple and yellow bars illustrate the calculated relative contributions of DMS (direct route) and SO_2_ oxidation to total H_2_SO_4_ formation at daytime. The contributions are calculated based on results from “no Cloud” simulation.

Process model simulations were performed with a complex multiphase chemistry mechanism MCM/CAPRAM^47,48^ for six different scenarios (Methods and Supplementary Table 2) to assess the importance of the direct gas-phase formation pathway of H_2_SO_4_ from DMS and CH_3_SH oxidation relative to the OH + SO_2_ reaction under pristine marine conditions. Oxidation of DMDS was neglected because of its relatively small emission^5^. The model is able to simulate typical DMS and SO_2_ mixing ratios (see Supplementary Figs. 10 and 11) as measured under marine conditions (Fig. 5a and attached Supplementary Dataset 1) independent of NO_x_ levels assumed in the simulations (Supplementary Fig. 12). The modelled CH_3_S and CH_3_SO_2_ formation rates are provided in Supplementary Table 3 for all six simulations. It can be seen that NO_x_ can likely affect the CH_3_S formation, but it is less important for CH_3_SO_2_ formation. The strongest impact on CH_3_SO_2_ production has the H_A_ applied. The modelled CH_3_S and CH_3_SO_2_ formation rates together with the experimental H_2_SO_4_ yields of 0.11% (CH_3_S), 1.2% (CH_3_SO_2_) and 100% (SO_2_) were used to calculate the gas-phase H_2_SO_4_ formation rates from the different oxidation pathways and their relative contributions (Fig. 5b). For more clarity, the simulations with higher NO_x_ are not depicted, because of the modelled low effect on CH_3_S and CH_3_SO_2_ formation rates in comparison with the simulation using the smaller NO emission. The data in Supplementary Table 3 reveal that the modelled CH_3_S and CH_3_SO_2_ formation rates are only weakly affected by ten times higher NO_x_ emissions, whereas the considered uptake parameters are the most important influencing factors. As the result, the direct gas-phase formation of H_2_SO_4_ arises mainly from the DMS addition channel and can contribute up to ∼50% to the overall gas-phase H_2_SO_4_ production. This emphasises the importance of the direct gas-phase formation route for marine conditions. Fully neglecting the share from the DMS addition channel because of inconsistent CH_3_SO_2_ yields currently in the literature^9,19,49^, still a fraction of up to ∼12% remains (Fig. 5b). It should be noted, that total direct gas-phase H_2_SO_4_ formation rates exclusively simulated by the model (Supplementary Fig. 13) exceeded those from the combined experiment/model approach (Fig. 5b) by about two orders of magnitude. A main reason for that is the inappropriate description of CH_3_SO_2_´s fate in the latest models^21,22^.

The simulations indicate that the concentration ratio of SO_2_ relative to the reduced-sulfur compounds, mainly DMS, is a critical parameter for the importance of the direct gas-phase H_2_SO_4_ formation. This becomes apparent using data from a field campaign at Baring Head, New Zealand^50^, with an air mass change from anthropogenically influenced, SO_2_/DMS > 10, to the clean pristine ocean regime, SO_2_/DMS < 0.1 (Fig. 5c). Significant relevance of the direct gas-phase H_2_SO_4_ route only exists for SO_2_/DMS ≤ 0.3. Thus, the direct gas-phase route could be important especially in the Southern Hemisphere, due to low SO_2_/DMS ratios existing there (Fig. 5a), and in the outflow of convective marine clouds where SO_2_ is reduced by scavenging and cloud chemistry.

Field studies often indicated new particle formation in the direct vicinity above marine clouds^51,52^ most likely connected to the high OH radical^53^ and H_2_SO_4_ concentrations^54^ observed at such locations. Updrafts of clouds can inject DMS into the free troposphere. There, we suggest that the direct gas-phase H_2_SO_4_ formation DMS → H_2_SO_4_, as identified in the present study, play an important role for gas-phase H_2_SO_4_ production in cloud outflows, because of the expected low SO_2_/DMS ratio immediately after cloud passage and the slow overall process DMS → SO_2_ → H_2_SO_4_. This implies that directly formed gas-phase H_2_SO_4_ from DMS oxidation is likely substantial for the observed new particle formation in cloud outflows, thereby affecting or even controlling the amounts of CCN available^51,52,55^. Therefore, this study provides the impetus for further developments to incorporate and study such processes in regional and global atmospheric chemistry transport as well as climate models.

In conclusion, we experimentally demonstrated the direct formation of H_2_SO_4_ in the course of atmospheric gas-phase oxidation of reduced-sulfur compounds. We found strong indications for the reactions CH_3_SO_2_ + O_3_ (pathway 9) and CH_3_SO_2_OO + RO_2_ (pathway 15) being the rate limiting steps for H_2_SO_4_ production under low-NO_x_ conditions. The strong increase of H_2_SO_4_ production in the presence of NO emphasises the role of CH_3_SO_2_OO radicals in this reaction system. Our findings do not support considerable MSA formation via the CH_3_SO_3_ + HO_2_ pathway.

Although the direct H_2_SO_4_ formation yields appear to be pretty small, for concentration ratios SO_2_/DMS ≤ 0.3, i.e. for conditions as encountered especially over the oceans in the Southern Hemisphere, and/or in the outflows of clouds, the direct route could be competitive with the established OH + SO_2_ path of H_2_SO_4_ generation.

All in all, we herewith suggest direct gas-phase formation of H_2_SO_4_ from reduced-sulfur compounds, such as DMS, to be an atmospherically relevant process for the production of H_2_SO_4_, and consequently for the formation of new particles, under, e.g. the pristine marine conditions in the Southern Hemisphere.

## Methods

### Experimental setup

The investigations were carried out in two flow systems, i.e. in the free-jet flow system^30,31^ and the laminar flow tube (LFT)^29^ at 1 bar of air and a temperature of 295 ± 2 K. The flow tubes worked with different residence times, 7.9 and 32 s, respectively, that allowed to draw a conclusion regarding the rate of relatively slow processes, here on the thermal decomposition of CH_3_SO_3_.

The free-jet flow system consists of an outer tube (length: 200 cm, inner diameter: 16 cm) and a moveable inner tube (outer diameter: 9.5 mm) connected with a nozzle of 3 mm inner diameter. The first reactant (isopropyl nitrite (IPN) or ozone) premixed with air (5 litre min^−1^, STP) was injected through the inner tube into the main gas stream (95 litre min^−1^, STP), which contained the second reactant (CH_3_SH and/or tetramethyl ethylene (TME) along with additions if needed) diluted in air. Downstream the nozzle, large differences of the gas velocities at the nozzle outflow (nozzle: 15.9 m s^−1^; main flow: 0.13 m s^−1^) and the nozzle geometry ensured rapid turbulent reactant mixing^56^. The reaction time was 7.9 s, experimentally obtained by a “chemical clock”. This set-up allows to carry out investigations under atmospheric conditions in absence of wall effects. IPN photolysis^57^ for continuous OH radical generation in the flow system was conducted downstream the mixing point of the reactants by means of 8 NARVA 36 W Blacklight Blue lamps emitting in the range 350–400 nm. The photolysis of IPN produces NO and i-C_3_H_7_O radicals, which rapidly formed acetone and HO_2_ radicals in the reaction with O_2_. OH radical generation finally took place via HO_2_ + NO → OH + NO_2_. Ozonolysis of TME^44^ served as non-photolytic OH radical source under low-NO_x_ conditions.

The laminar flow tube (LFT) (i.d. 8 cm; total length 425 cm) consists of a first section (56 cm) containing the gas inlet system, a second middle section (344 cm) representing the reaction zone and an end section (25 cm) incorporating the sampling devices. Here, TME ozonolysis was exclusively used for OH radical formation. Ozone was injected through a nozzle system into the gas mixture, containing TME, reduced-sulfur compounds and additives if needed, just before entering the middle section. The total flow was set at 30 litre min^−1^ (STP) resulting in a residence time of 32 s in the reaction zone.

Humidified air in both setups was supplied by flushing a part of the air flow through three water saturators filled with water from an ultrapure water system (Barnstead, resistivity: 17.4 MΩ cm). The relative humidity of the reaction gas was continuously controlled at the outflow by a humidity sensor (Hygrosense HYTE). Ozone was monitored by a gas monitor (Thermo Scientific iQ 49) and the concentration of organic compounds by a proton transfer reaction - mass spectrometer (Ionicon, high sensitivity PTR-MS)^58^. The “crude” air was taken from a pressure swing adsorption unit with further purification by means of absorber units filled with charcoal, a hopcalite (CuMnO_x_) catalyst^59^ and different activated 4 Å and 10 Å molecular sieves.

### Reactant concentrations and conversion and the importance of OH + SO_2_ and Criegee intermediate + SO_2_

Initial reactant concentrations are either given in Supplementary Table 1 for the experiments described in the main text, Figs. 2–4, or in the figure captions. The amount of reacted CH_3_SH in the IPN photolysis experiment (Fig. 2a) was measured in an additional run in the presence of SO_2_ (for otherwise identical reaction conditions) by monitoring H_2_SO_4_ formation. The SO_2_ concentration, 7.5 × 10^11^ molecules cm^−3^, was chosen to such an extent that only 2% of formed OH radicals reacted with SO_2_ and, thus, the product formation of the OH + CH_3_SH reaction was not disturbed^12^. Reacted CH_3_SH is available from the measured H_2_SO_4_ (after correction of the fraction arising from CH_3_SH oxidation) considering the OH reactivity in the parallel reactions OH + CH_3_SH and OH + SO_2_. In the case of TME ozonolysis for OH production, the amount of reacted CH_3_SH or DMDS was calculated based on a detailed reaction scheme (Supplementary Table 4). Modelling calculations, including the IPN photolysis experiment, confirmed that H_2_SO_4_ production starting from the reaction of SO_2_ with OH radicals or Criegee intermediates did not significantly influence the results of direct H_2_SO_4_ formation from the organic sulfur compounds.

### Mass spectrometric analysis

Detection of H_2_SO_4_, CH_3_SO_3_H and other oxidation products was carried out using a CI-APi-TOF (chemical ionisation - atmospheric pressure interface - time-of-flight) mass spectrometer with a resolving power > 3000 Th/Th (Tofwerk) connected to a Boulder-type inlet system (Airmodus) operating with iodide (I^-^) and nitrate (NO_3_^-^) as the reagent ions at atmospheric pressure^12,31^.

In the case of ionisation by iodide, tert-butyl iodide premixed in a flask was added to a 35 litre min^−1^ (STP) sheath flow of purified nitrogen leading to a tert-butyl iodide concentration of 4.8 × 10^11^ molecules cm^−3^. Produced ions after ionisation with a ^241^Am source were I^-^ and to a lesser amount I(H_2_O)^-^. The ions from the sheath flow were guided into the sample flow by an electric field without mixing of both gas streams. In the case of ionisation by nitrate, an HNO_3_ containing vial was connected to the 35 litre min^−1^ (STP) flow without overflowing the HNO_3_ sample. HNO_3_ diffusion from the vial was found to be sufficient to form the reagent ions (HNO_3_)_x_NO_3_^-^, x = 0, 1, 2, after ionisation.

Absolute signal calibration was used in the measurements of H_2_SO_4_ applying iodide and nitrate ionisation as well as in the determination of SO_3_, which was detected as the adduct (SO_3_)NO_3_^-^ and SO_4_^-^ ^60,61^ using nitrate ionisation. H_2_SO_4_ and SO_3_ production in the calibration experiments for wet (r.h. = 10%) and dry (r.h. <0.1%) conditions, respectively, was carried out via TME ozonolysis in the presence of SO_2_^62^. The calibration factors obtained for H_2_SO_4_ were also taken for CH_3_SO_3_H. In the case of CH_3_SO_2_, CH_3_SO_2_OOH and CH_3_SO_2_OONO_2_, a calculated calibration factor of 2 × 10^9^ molecules cm^−3^ was taken, resulting in lower limit concentrations for these products with an uncertainty of a factor of two^31,45^.

### Kinetic data analysis

H_2_SO_4_ and MSA wall loss in the LFT:

The rate law for H_2_SO_4_ is given by

1 $$\frac{d\left[{{H}_{2}{SO}}_{4}\right]}{{dt}}=\,{P}_{{{H}_{2}{SO}}_{4}}-{k}_{{loss}}\, \times \,[{{H}_{2}{SO}}_{4}]$$

assuming a time-independent production term of H_2_SO_4_, ${P}_{{{H}_{2}{SO}}_{4}}$. This assumption is justified because of constant OH radical production during the whole reaction time and practically constant reactant concentrations due to reactant conversions clearly smaller than 1% in each case. Integration of Eq. (1) with ${[{{H}_{2}{SO}}_{4}]}_{t=0}$ = 0 yields:

2 $${[{{H}_{2}{SO}}_{4}]}_{t}=\frac{{P}_{{{H}_{2}{SO}}_{4}}}{{k}_{{loss}}}\,\left(1-\exp \left({-k}_{{loss}}\,{{\times }}\,t\right)\right)$$

${[{{H}_{2}{SO}}_{4}]}_{t}={P}_{{{H}_{2}{SO}}_{4}}{\times }{t}$ follows for the wall-loss free H_2_SO_4_ concentration. Consequently, the relative *H*_*2*_*SO*_*4*_ *loss* in the tube is given by:

3 $${{H}_{2}{SO}}_{4}\,{loss}=1-\frac{1}{{k}_{{loss}}\,{{\times }}\,t}\left(1-\exp \left({-k}_{{loss}}\,{{\times }}\,t\right)\right)$$

The value of ${k}_{{loss}}$ can be described by the diffusion-controlled wall-loss term $\frac{3.65{x\;D}}{{r}^{2}}$ using an experimentally obtained H_2_SO_4_ diffusion coefficient D = 0.08 cm^2^ s^−1^ ^63^ leading to ${k}_{{loss}}$ = 0.018 s^−1^. Based on that and for the reaction time of 32 s in the LFT, a H_2_SO_4_ loss of 24% was calculated using Eq. (3). Thus, the measured H_2_SO_4_ concentration was multiplied with 1.315 to consider the wall loss. The same was applied for MSA.

Determination of *k*_*17*_ describing CH_3_SO_3_ → SO_3_ + CH_3_:

Assuming dominant loss of CH_3_SO_3_ via its decomposition into SO_3_ and CH_3_ and a time-independent production term of CH_3_SO_3_, ${P}_{{{{CH}}_{3}{SO}}_{3}}$, due to practically constant reactant conditions, the rate law of CH_3_SO_3_ is given by:

4 $$\frac{d\left[{{{CH}}_{3}{SO}}_{3}\right]}{{dt}}=\,{P}_{{{{CH}}_{3}{SO}}_{3}}-{k}_{17}\, \times \,[{{{CH}}_{3}{SO}}_{3}]$$

Integration of Eq. (4) with ${[{{{CH}}_{3}{SO}}_{3}]}_{t=0}$ = 0 yields:

5 $${[{{{CH}}_{3}{SO}}_{3}]}_{t}=\frac{{P}_{{{{CH}}_{3}{SO}}_{3}}}{{k}_{17}}\,\left(1-\exp \left({-k}_{17}\,{{\times }}\,t\right)\right)$$

The rate law of SO_3_ formation is

6 $$\frac{d[S{O}_{3}]}{dt} 	={k}_{17}{\times }[C{H}_{3}S{O}_{3}]\\ 	={P}_{C{H}_{3}S{O}_{3}}{\times }(1-\exp (-{k}_{17}{\times }t))$$

leading after integration with ${[{{SO}}_{3}]}_{t=0}$ = 0 to:

7 $$\frac{{[{{SO}}_{3}]}_{t}}{{P}_{{{{CH}}_{3}{SO}}_{3}}}=t-\,\frac{1}{{k}_{17}}\,\left(1-\exp \left({-k}_{17}\,{{\times }}\,t\right)\right)$$

Because of immediate SO_3_ conversion to H_2_SO_4_ under humid conditions, Eq. (7) can be written in the following way:

8 $$\frac{{[{{H}_{2}{SO}}_{4}]}_{t}}{{P}_{{{{CH}}_{3}{SO}}_{3}}}=t-\,\frac{1}{{k}_{17}}\,\left(1-\exp \left({-k}_{17}\,{{\times }}\,t\right)\right)$$

Equation (8) was used to determine ${k}_{17}$ based on measured H_2_SO_4_ concentrations from OH + CH_3_SH depending on NO_2_ additions in both flow systems, i.e. in the free-jet flow system with t = 7.9 s and in the LFT with t = 32 s for otherwise similar conditions (Supplementary Fig. 14 and Fig. 4d). The ratio ${[{{{{{{{\rm{H}}}}}}}_{2}{{{{{\rm{SO}}}}}}}_{4}]}_{32{{{{{\rm{s}}}}}}}$/${[{{{{{{{\rm{H}}}}}}}_{2}{{{{{\rm{SO}}}}}}}_{4}]}_{7.9{{{{{\rm{s}}}}}}}$ was found to be 4.5 ± 0.6 with a corresponding ratio of the CH_3_SO_3_ production rates ${P}_{{{{CH}}_{3}{SO}}_{3}}$ of 1/2.27, that considered the different reactant concentrations in the experiments and the fraction of OH radicals reacting with CH_3_SH. For the mean H_2_SO_4_ ratio of 4.5, ${k}_{17}$ = 0.076 s^−1^ was calculated leading to ${0.076}_{-0.025}^{+0.034}$ s^−1^ that involves the bounds of the H_2_SO_4_ ratio.

Based on Eq. (9), which corresponds to Eq. (3) for H_2_SO_4_ loss,

9 $${{{CH}}_{3}{SO}}_{3}\,{decomposition}=1-\frac{1}{{k}_{17}\,{{\times }}\,t}\left(1-\exp \left({-k}_{17}\,{{\times }}\,t\right)\right)$$

it was possible to calculate with ${k}_{17}$ = 0.076 s^−1^, that 62% of formed CH_3_SO_3_ decomposed in the LFT (t = 32 s) making a correction factor of 1.6 necessary in order to account for total removal via pathway 17. It is to be noted, that the reaction of CH_3_SH with CH_3_SO_3_, pathway 19, does not influence this result as long as the contribution of this pathway (same CH_3_SH concentration) is identical in both flow experiments.

### Atmospheric modelling

Complex multiphase chemistry simulations were performed using the SPectral Aerosol Cloud Chemistry Interaction Model (SPACCIM)^64^ to study the contributions of different reaction pathways from DMS, SO_2_ and CH_3_SH leading to sulfuric acid or its precursors under pristine marine conditions. It should be noted, that the applied model is not designed to simulate new particle formation. Thus, nucleation driven by gas-phase H_2_SO_4_ and resulting effects cannot be investigated. Therefore, we only focus on the chemical gas-phase H_2_SO_4_ formation in the present study.

In the model, the multiphase chemistry is described by combining the near-explicit gas-phase mechanism MCMv3.2^65,66^ and detailed aqueous-phase chemistry mechanism CAPRAM4.0 (Chemical Aqueous Phase RAdical Mechanism version 4.0)^47^, respectively. This mechanism system describes the formation of gas-phase H_2_SO_4_ and aqueous sulfate in a very detailed manner. The representation of the specific multiphase chemistry of reactive halogen species and dimethyl sulfide, important for the marine atmosphere, is achieved through two additional reaction modules, CAPRAM–HM3.0^48^ and CAPRAM–DM1.0^21^. With these two additional modules, CAPRAM4.0 includes almost all known sulfate formation pathways in the atmospheric aqueous phase, such as S(IV) oxidation by H_2_O_2_, O_3_, HNO_4_, reactive halogen species (X_2_^-^ radical or HOX, with X = Cl, Br or I) or transition metal ions.

Due to the intended foci of the simulations, the complex multiphase DMS chemistry scheme of CAPRAM-DM1.0 has been upgraded with recent mechanism updates and an oxidation scheme for CH_3_SH was implemented (Supplementary Table 5). The mechanistic updates comprise the formation of the hydroperoxymethyl thioformate (HPMTF) and its further oxidation in the gas phase. The gas-phase HPMTF oxidation follows mainly the proposed routes described by Wu et al. (2015)^11^, considering SO_2_ or OCS formation, only. Thus, HPMTF cannot contribute to the direct gas-phase formation of H_2_SO_4_ in this mechanism. Phase transfer and subsequent aqueous-phase processing of HPMTF, not included yet because of the current high uncertainties, do not change this. Briefly, the revised mechanism scheme contains 128 gas-phase reactions, 5 phase transfer processes and 50 aqueous-phase reactions.

In the process simulations, an aerosol particle spectrum representative for pristine marine conditions is included^67^. The whole model setup (emission, deposition, initialisation of the gas-phase and particle-phase composition) is the same as applied in previous DMS chemistry studies^21,48^. An exception is the newly included emission of CH_3_SH (emission rate: 3.18 × 10^3^ molecules cm^−3^ s^−1^), which is a factor of ten lower compared to that of DMS. This difference is in line with field measurements, see attached Supplementary Dataset 1.

In total, six simulations were performed, separated into (i) three with (“Cloud”) and (ii) three without (“no Cloud”) cloud processing along the air parcel trajectory. The total simulation time is 108-hours but only day 2 to 4 were considered for data analysis to avoid spin-up effects. Runs are performed for summer conditions, with a boundary layer temperature and relative humidity of 280 K and 70% during non-cloud periods. In the simulations with cloud interactions, eight non-permanent clouds are considered. Every cloud exists for about two hours and occurs around noon and midnight, respectively. Cloud formation is achieved through adiabatic cooling of the air parcel 15 minutes before 11 a.m. and p.m., and the cloud evaporation is realised by adiabatic warming 15 minutes after 1 p.m. and a.m., respectively. Besides the two microphysical scenarios (“Cloud”, “no Cloud”), simulations were run with Henry’s Law constants implemented in the base mechanism^21^ and with lower Henry’s Law constants for DMSO (H_A,298K_ = 2.43 × 10^5^ mol atm^−1^), DMSO_2_ (H_A,298K_ = 1.18 × 10^6^ mol atm^−1^), and MSIA (H_A,298K_ = 1.69 × 10^6^ mol atm^−1^) calculated by COSMOtherm^22^. The two uptake cases were performed to consider the potential uncertainty in the Henry’s Law constants and to study their impact of the sulfuric acid formation. In addition, simulations with an increased NO emission by a factor of ten were run using Henry’s Law constants implemented in the base mechanism. All simulation scenarios together with their individual configurations are outlined in Supplementary Table 2.

For the six different simulations, averaged net rates (in molecules cm^−3^ s^−1^) for the daytime oxidation of DMS, CH_3_SH and SO_2_ between the second to the fourth model day were calculated as well as primary daytime production rates of CH_3_S and CH_3_SO_2_. For the oxidation of DMS, we distinguish between rates of the addition and abstraction pathways. All calculated rates are given in Supplementary Table 3.

### Supplementary information

Supplementary Information

Peer Review File

Description of Additional Supplementary Files

Supplementary Data 1

### Source data

Source Data

## Data Availability

The measurement data collected from the literature and used in this work are provided in the attached Supplementary Dataset 1. The data generated in this study are provided in the Supplementary Information. Source data are provided with this paper.

## Code availability

The code of MCM is provided via http://mcm.leeds.ac.uk/MCM and CAPRAM code is available at https://capram.tropos.de/. The new and updated mechanism implementation data are provided in the Supplementary Information.
