# Supplementary material for: Direct sulfuric acid formation from the gas-phase oxidation of reduced-sulfur compounds
Source: Nat Commun. 2023 Aug 10;14:4849. doi: 10.1038/s41467-023-40586-2 (PMC10415363; doi:10.1038/s41467-023-40586-2)
Supplement: Supplementary file 1 — Supplementary Information [file 41467_2023_40586_MOESM1_ESM.pdf]

Supplementary Information for

Direct sulfuric acid formation from the gas-phase oxidation of reduced-sulfur compounds

Torsten Berndt[1]*, Erik H. Hoffmann[1], Andreas Tilgner[1], Frank Stratmann[2] & Hartmut Herrmann[1]

[1] Atmospheric Chemistry Department (ACD), Leibniz Institute for Tropospheric Research (TROPOS), 04318 Leipzig, Germany.
[2] Atmospheric Microphysics Department (AMP), Leibniz Institute for Tropospheric Research (TROPOS), 04318 Leipzig, Germany.

Corresponding author: Torsten Berndt, e-mail: berndt@tropos.de

The PDF file includes:

Tables S1 to S5
Supplementary Figs. 1 to 14
Supplementary References

Supplementary Tables

Supplementary Table 1: Experimental conditions of investigations described in the main text.

**Experiment description**	**Reactants** (molecules cm^{-3})	**Relative humidity** (%)	**OH source**	**Ionisation technique**	**Plot**
Overview about product formation in the free-jet flow system	$[CH_3SH] = 6.6 \times 10^{11}$<br>$[IPN] = (2.0 - 20) \times 10^{11}$	10	IPN photolysis	iodide	Fig. 2
Impact of CH_3SH on the formation of H_2SO_4 and MSA, free-jet flow system	$[CH_3SH] = (6.7 - 202) \times 10^{10}$<br>$[IPN] = 4.0 \times 10^{11}$<br>$[NO] = 1.0 \times 10^{10}$	10	IPN photolysis	nitrate	Fig. 3
Impact of ozone on the formation of H_2SO_4 or SO_3 (dry conditions), laminar flow tube (LFT)	$[CH_3SH] = 2.0 \times 10^{11}$<br>$[CH_3SSCH_3] = 6.1 \times 10^{10}$<br>$[TME] = 5.0 \times 10^{9}$<br>$[O_3] = (1.5 - 13) \times 10^{11}$	10 or < 0.1	O_3 + TME	nitrate	Fig. 4a
Impact of RO_2 radicals on the formation of H_2SO_4 and MSA, LFT	$[CH_3SH] = 2.0 \times 10^{11}$<br>$[O_3] = 5.7 \times 10^{11}$<br>$[TME] = 0.5, 5.0, or 10 \times 10^{10}$<br>$[CH_4] = 0, 1.0 or 2.0 \times 10^{16}$	10	O_3 + TME	nitrate	Fig. 4b
Impact of NO on H_2SO_4 formation, free-jet flow system or LFT	$[CH_3SH] = 2.0 \times 10^{11}$<br>$[CH_3SSCH_3] = 6.1 \times 10^{10}$<br>$[TME] = 5.0, 15 or 50 \times 10^{9}$<br>$[O_3] = 5.7 \times 10^{11}$<br>$[NO] = (7.8 - 1000) \times 10^{8}$	10	O_3 + TME	nitrate	Fig. 4c
Impact of NO_2 on H_2SO_4 formation, free-jet flow system or LFT	$[CH_3SH] = 2.0 \times 10^{11}$<br>$[CH_3SSCH_3] = 6.1 \times 10^{10}$<br>$[TME] = 5.0, 15 or 50 \times 10^{9}$<br>$[O_3] = 5.7 \times 10^{11}$<br>$[NO_2] = (3.3 - 100) \times 10^{9}$	10	O_3 + TME	nitrate	Fig. 4d

Supplementary Table 2: Overview of the specification for the different simulations.

Simulation case	Microphysical conditions	Remarks
Cloud	8 cloud passages (4 daytime clouds between 11 a.m. to 1 p.m. and 4 night-time clouds between 11p.m. to 1 a.m.)	
Cloud, lower H_A	8 cloud passages (4 daytime clouds between 11 a.m. to 1 p.m. and 4 night-time clouds between 11p.m. to 1 a.m.)	as "Cloud" but with lower Henry's Law constants ($H_{A, 298K}$) for DMSO, $DMSO_2$ and MSIA based on theoretical method used by Wollesen de Jonge et al. (2021)[1]
no Cloud	no cloud passages	
no Cloud, lower H_A	no cloud passages	as "no Cloud" but with lower Henry's Law constants ($H_{A, 298K}$) for DMSO, $DMSO_2$ and MSIA based on theoretical method used by Wollesen de Jonge et al. (2021)[1]
Cloud with higher NO_x	as "Cloud"	as "Cloud" but with ten times higher NO emissions
no Cloud with higher NO_x	as "no Cloud"	as "no Cloud" but with ten times higher NO emissions

Supplementary Table 3: Day 2-4 averaged rates of daytime DMS, CH_3SH and SO_2 oxidation (red) and primary daytime production rates of CH_3S and CH_3SO_2 (blue) of the different simulations. The **bold** values refer towards the molar yields from DMS or CH_3SH oxidation, respectively. Normalized reaction rates (k_{1st}) are calculated from the averaged net reaction rates divided by the modeled concentrations of DMS, CH_3SH and SO_2, respectively.

	**DMS Oxidation**		**CH_3SH Oxidation**	**OH + SO_2**
	Addition / molec. cm^{-3} s^{-1}	*Abstraction /* molec. cm^{-3} s^{-1}	molec. cm^{-3} s^{-1}	molec. cm^{-3} s^{-1}
**Cloud**				
Total daytime 2-4 average rate (avg. k_{1st})	4.8×10^{4} (7.9×10^{-6} s^{-1})	1.9×10^{4} (3.3×10^{-6} s^{-1})	8.0×10^{3} (1.6×10^{-5} s^{-1})	7.0×10^{1} (1.6×10^{-6} s^{-1})
Daytime 2-4 average rate of primarily formed CH_3S	–	4.1×10^{3} **22%**	8.0×10^{3} **100%**	
Daytime 2-4 average rate of primarily formed CH_3SO_2	2.1×10^{3} **4%**	–	–	
**Cloud with ten times higher NO emission rate**				
Total daytime 2-4 average rate (avg. k_{1st})	4.8×10^{4} (8.8×10^{-6} s^{-1})	2.2×10^{4} (4.1×10^{-6} s^{-1})	8.2×10^{3} (1.8×10^{-5} s^{-1})	9.2×10^{1} (2.1×10^{-6} s^{-1})
Daytime 2-4 average rate of primarily formed CH_3S	–	4.1×10^{3} **19%**	8.2×10^{3} **100%**	
Daytime 2-4 average rate of primarily formed CH_3SO_2	2.4×10^{3} **5%**	–	–	
**Cloud, lower H_A ($H_{A,\,298K}$ from Wollesen de Jonge et al., 2021)[1]**				
Total daytime 2-4 average rate (avg. k_{1st})	4.8×10^{4} (7.9×10^{-6} s^{-1})	1.9×10^{4} (3.3×10^{-6} s^{-1})	8.0×10^{3} (1.6×10^{-5} s^{-1})	7.4×10^{1} (1.2×10^{-6} s^{-1})
Daytime 2-4 average rate of primarily formed CH_3S	–	4.2×10^{3} **22%**	8.0×10^{3} **100%**	
Daytime 2-4 average rate of primarily formed CH_3SO_2	7.3×10^{3} **15%**	–	–	

**no Cloud**				
Total daytime 2-4 average rate (avg. k_{1st})	5.1×10^4 (9.2×10^{-6} s^{-1})	2.5×10^4 (4.5×10^{-6} s^{-1})	8.5×10^3 (2.1×10^{-5} s^{-1})	1.2×10^3 (4.6×10^{-7} s^{-1})
Daytime 2-4 average rate of primarily formed CH_3S	–	6.8×10^3 **27%**	8.5×10^3 **100%**	
Daytime 2-4 average rate of primarily formed CH_3SO_2	4.6×10^3 **9%**	–	–	
**no Cloud with ten times higher NO emission rate**				
Total daytime 2-4 average rate (avg. k_{1st})	4.8×10^4 (9.7×10^{-6} s^{-1})	2.8×10^4 (5.7×10^{-6} s^{-1})	8.5×10^3 (2.1×10^{-5} s^{-1})	1.4×10^3 (5.0×10^{-7} s^{-1})
Daytime 2-4 average rate of primarily formed CH_3S	–	8.7×10^3 **31%**	8.5×10^3 **100%**	
Daytime 2-4 average rate of primarily formed CH_3SO_2	4.7×10^3 **10%**	–	–	
**no Cloud, lower H_A ($H_{A, 298K}$ from Wollesen de Jonge et al., 2021)[1]**				
Total daytime 2-4 average rate (avg. k_{1st})	5.1×10^4 (9.0×10^{-6} s^{-1})	2.4×10^4 (4.4×10^{-6} s^{-1})	8.4×10^3 (2.1×10^{-5} s^{-1})	1.3×10^3 (4.4×10^{-7} s^{-1})
Daytime 2-4 average rate of primarily formed CH_3S	–	6.6×10^3 **28%**	8.4×10^3 **100%**	
Daytime 2-4 average rate of primarily formed CH_3SO_2	2.7×10^4 **53%**	–	–	

Supplementary Table 4: Reaction scheme describing processes in the flow experiments. Rate coefficients at ~295 K were taken from literature or have been estimated. Further reactions of CH_3SO_3 as well as CH_3SO_2 + O_2 and subsequent steps were not considered to simplify matters.

**Reaction**	**Rate coefficient (cm^3 $molecule^{-1}$ s^{-1} or s^{-1})**
IPN (+O_2) $\rightarrow$ NO + HO_2 + acetone	0.0016
O_3 + TME $\rightarrow$ 0.4×sCI + 0.52×OH + 0.08×HO_2 + 0.52×$CH_3C(O)CH_2O_2$	1.0×10^{-15} [2]
OH + TME $\rightarrow$ HO-TMEO_2	1.1×10^{-10} [3]
NO + HO_2 $\rightarrow$ OH + NO_2	8.9×10^{-12} [3]
OH + HO_2 $\rightarrow$ H_2O + O_2	1.1×10^{-10} [3]
HO_2 + HO_2 $\rightarrow$ H_2O_2 + O_2	1.65×10^{-12} [3]
OH + NO $\rightarrow$ HNO_2	1.0×10^{-11} [3]
OH + NO_2 $\rightarrow$ HNO_3	1.2×10^{-11} [3]
O_3 + NO $\rightarrow$ NO_2 + O_2	1.8×10^{-14} [3]
O_3 + NO_2 $\rightarrow$ NO_3 + O_2	3.5×10^{-17} [3]
O_3 + HO_2 $\rightarrow$ OH + 2×O_2	2.0×10^{-15} [3]
OH + CH_3SH $\rightarrow$ CH_3S + H_2O	3.3×10^{-11} [4]
OH + CH_3SSCH_3 $\rightarrow$ CH_3S + CH_3SOH	2.3×10^{-10} [4]
OH + SO_2 (+O_2) $\rightarrow$ SO_3 + HO_2	8.9×10^{-13} [4]
OH + CH_4 (+O_2) $\rightarrow$ CH_3O_2 + H_2O	6.28×10^{-15} [5]
sCI $\rightarrow$ OH + $CH_3C(O)CH_2O_2$	900[6]
sCI + SO_2 $\rightarrow$ SO_3 + …	1.55×10^{-10} [7]
sCI + NO_2 $\rightarrow$ NO_3 + …	2.1×10^{-12} [7]
O_3 + CH_3SOH $\rightarrow$ CH_3SO_2 + HO_2	2.0×10^{-12} [8]
HO-TMEO_2 + NO $\rightarrow$ 0.9×NO_2 + 0.9×HO_2	8.8×10^{-12} [9]
$CH_3C(O)CH_2O_2$ + NO $\rightarrow$ 0.9×NO_2 + 0.9×HO_2	8.8×10^{-12} [9]
CH_3O_2 + NO $\rightarrow$ 0.9×NO_2 + 0.9×HO_2	7.7×10^{-12} [10]
HO-TMEO_2 + HO_2 $\rightarrow$ products	1.0×10^{-11} [estimated]
$CH_3C(O)CH_2O_2$ + HO_2 $\rightarrow$ products	9.0×10^{-12} [5]
CH_3O_2 + HO_2 $\rightarrow$ products	5.2×10^{-12} [5]

$CH_3C(O)CH_2O_2 + CH_3C(O)CH_2O_2 \rightarrow$ products	8.0×10^{-12} [11]
$CH_3C(O)CH_2O_2$ + HO-TMEO$_2$ $\rightarrow$ products	1.0×10^{-13} [estimated]
HO-TMEO$_2$ + HO-TMEO$_2$ $\rightarrow$ products	1.1×10^{-14} [12]
$CH_3S + O_3 \rightarrow CH_3SO + O_2$	4.9×10^{-12} [4]
$CH_3SO + O_3 (+O_2) \rightarrow SO_2 + CH_3O_2 + O_2$	6.0×10^{-13} [13]
$CH_3S + O_2 \rightarrow CH_3SOO$	2.5×10^{-14} [14]
$CH_3SOO \rightarrow CH_3S + O_2$	2.0×10^{5} [14]
$CH_3SOO \rightarrow CH_3SO_2$	5 [estimated]
$CH_3SO_2 (+O_2) \rightarrow SO_2 + CH_3O_2$	20 [estimated]
$CH_3SO_2 + O_3 \rightarrow CH_3SO_3 + O_2$	3.0×10^{-13} [15]
$CH_3S + NO_2 \rightarrow CH_3SO + NO$	6.1×10^{-11} [4]
$CH_3SO + NO_2 \rightarrow 0.5\times SO_2 + 0.5\times CH_3O_2 + 0.5\times CH_3SO_2 + NO$	1.2×10^{-11} [4]
$CH_3SOO + NO \rightarrow CH_3SO + NO_2$	1.2×10^{-11} [4]
$CH_3SO_2 + NO_2 \rightarrow CH_3SO_3 + NO$	4.0×10^{-12} [15]
OH $\rightarrow$ wall *	0.053 §
$HO_2 \rightarrow$ wall *	0.045 §
$CH_3S \rightarrow$ wall *	0.018 §
$CH_3SOO \rightarrow$ wall *	0.018 §
$CH_3SO \rightarrow$ wall *	0.018 §
$CH_3SO_2 \rightarrow$ wall *	0.018 §
$CH_3O_2 \rightarrow$ wall *	0.032 §
$CH_3C(O)CH_2O_2 \rightarrow$ wall *	0.018 §
HO-TMEO$_2$ $\rightarrow$ wall *	0.018 §

* only in the LFT

§ diffusion-limited rate coefficient calculated for the LFT

Supplementary Table 5: Overview on the new implemented (termed new) or updated reactions in CAPRAM-DM1.0 in both, gas and aqueous phase. Second order rate coefficients are given in cm^3 $molecules^{-1}$ s^{-1} or l mol^{-1} s^{-1} for gas or aqueous phase, respectively, and first order rate coefficients in s^{-1}.

No.	Reaction	Rate coefficient at ~295 K (if not otherwise stated)	Comment
**Gas-phase reactions**			
gD52	$CH_3S(O)OH + OH \rightarrow CH_3SO_2 + H_2O$	9.0×10^{-11}	forming solely CH_3SO_2
new	$CH_3SCH_2O_2 \rightarrow HOOCH_2SCH_2O_2$	$2.74\times10^{7}exp(-5950/T)$	Ref.[16]
new	$HOOCH_2SCH_2O_2 + HO_2 \rightarrow HOOCH_2SCH_2OOH + O_2$	$1.91\times10^{-13}exp(1300/T)$	MCMv3.2 [17]
new	$HOOCH_2SCH_2O_2 + NO \rightarrow HOOCH_2SCH_2O + NO2$	$4.90\times10^{-12}exp(260/T)$	MCMv3.2 [17]
new	$HOOCH_2SCH_2O_2 + NO_3 \rightarrow HOOCH_2SCH_2O + NO_2$	2.30×10^{-12}	MCMv3.2 [17]
new	$HOOCH_2SCH_2O_2 \rightarrow 0.8\ HOOCH_2SCH_2O + 0.1\ HOOCH_2SCH_2OH + 0.1\ HOOCH_2SCHO$	3.74×10^{-12}	MCMv3.2 [17]
new	$HOOCH_2SCH_2O_2 \rightarrow HOOCH_2SCHO + OH$	$4.20\times10^{7}exp(-5390/T)$	Ref.[16]
new	$HOOCH_2SCH_2O + O_2 \rightarrow HOOCH_2SCHO + HO_2$	$2.50\times10^{-14}exp(-300/T)$	est. MCMv3.2 [17]
new	$HOOCH_2SCH_2O \rightarrow HOOCH_2S + HCHO$	1.00×10^{6}	Ref.[18]
new	$HOOCH_2SCH_2OH + OH \rightarrow HOOCH_2SCHO + HO_2$	2.78×10^{-11}	as for CH_3SCH_2OH in MCMv3.2 [17]
new	$HOOCH_2SCH_2OOH + h\nu \rightarrow HOOCH_2SCH_2O$	$J=1.530\times10^{-5}cos(\chi)^{0.682}exp(-0.279 / cos(\chi))$	as for CH_3OOH
new	$HOOCH_2SCH_2OOH + OH \rightarrow HOOCH_2SCH_2O_2 + H_2O$	$7.63\times10^{-13}exp(635/T)$	Ref.[19]
new	$HOOCH_2SCH_2OOH + OH \rightarrow HOOCH_2SCHO + OH + H_2O$	7.03×10^{-11}	as for CH_3SCH_2OOH in MCMv3.2 [17]
new	$HOOCH_2SCHO + h\nu \rightarrow HCHO + OCS + HO_2 + OH$	$J=7.649\times10^{-6}cos(\chi)^{0.682}exp(-0.279 / cos(\chi))$	as for CH_3OOH in MCMv3.2 [17]
new	$HOOCH_2SCHO + h\nu \rightarrow HOOCH_2S + CO + HO_2$	$J=2.792\times10^{-5}cos(\chi)^{0.805}exp(-0.338 / cos(\chi))$	as for CH_3SCHO in MCMv3.2 [17]
new	$HOOCH_2SCHO + OH \rightarrow HOOCH_2SCO$	1.40×10^{-12}	Ref.[18]
new	$HOOCH_2SCO \rightarrow OH + HCHO + OCS$	$9.20\times10^{9}exp(-505.4/T)$	Ref.[18]
new	$HOOCH_2SCO \rightarrow HOOCH_2S + CO$	$1.60\times10^{9}exp(-1468.6/T)$	Ref.[18]
new	$HOOCH_2S + O_3 \rightarrow HOOCH_2SO + O_2$	$1.15\times10^{-12}exp(430/T)$	Ref.[18]
new	$HOOCH_2S + NO_2 \rightarrow HOOCH_2SO + NO$	$6.00\times10^{-11}exp(240/T)$	Ref.[18]
new	$HOOCH_2SO + O_3 \rightarrow SO_2 + HCHO + OH + O_2$	4.00×10^{-13}	Ref.[18]

new	$HOOCH_2SO + NO_2 \rightarrow$ $SO_2 + HCHO + OH + NO$	1.20×10^{-11}	Ref.[18]
new	$CH_3SH + OH \rightarrow CH_3S + H_2O$	$9.9\times10^{-12}exp(356/T)$	Ref.[4]
new	$CH_3SH + NO_3 \rightarrow CH_3S +$ HNO_3	9.2×10^{-13}	Ref.[4]
new	$CH_3SH + Cl \rightarrow CH_3S + HCl$	$1.2\times10^{-10}exp(150/T)$	Ref.[4]
new	$CH_3SH + BrO \rightarrow CH_3S +$ $HOBr$	$2.2\times10^{-15}exp(827/T)$	Ref.[20]
**Aqueous-phase reaction**			
new	$CH_3S(O)(Br)CH_3 + H_2O \rightarrow$ $CH_3S(O)OH + HBr + CH_3$	1.0×10^{7}	est. as for $CH_3S(O)(Cl)CH_3$[21]

Supplementary Figures

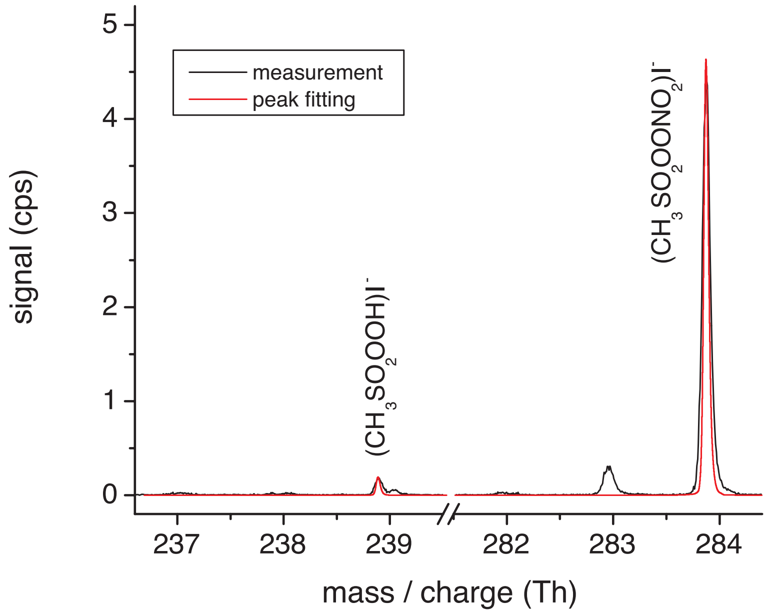

Supplementary Fig. 1: Product spectrum of CH_3S oxidation (in part). Observed raw spectrum in the range 236.6 - 239.5 Th and 281.5 - 284.4 Th from 10 min data accumulation compared with the calculated signals of iodide adducts with CH_3SO_2OOH and $CH_3SO_2OONO_2$ from peak fitting. The experiment was carried out in the free-jet flow system with r.h. = 10% using IPN photolysis for OH production. Reactant concentrations were $[CH_3SH] = 6.6 \times 10^{11}$ and $[IPN] = 2.0 \times 10^{12}$ molecules cm^{-3} and a reaction time of 7.9 s. Source data are provided as a Source Data file.

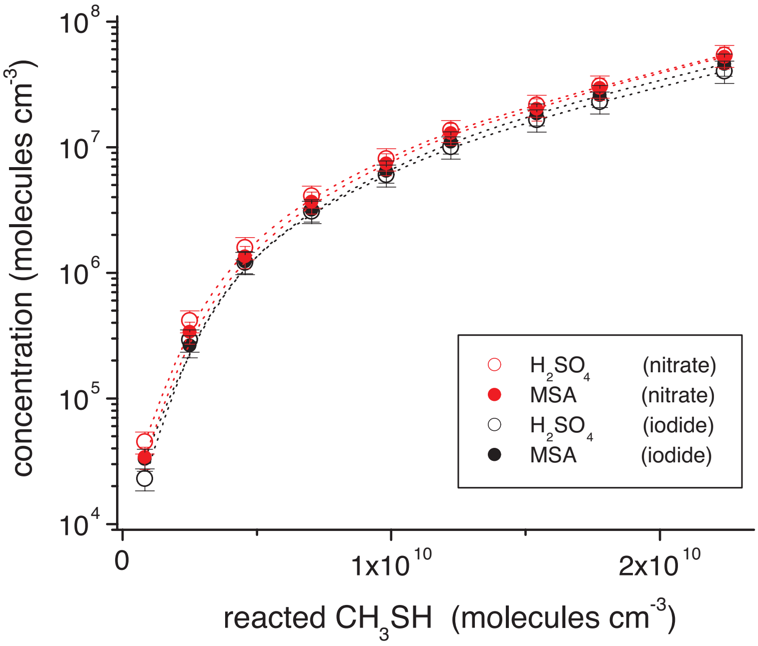

Supplementary Fig. 2: Formation of H_2SO_4 and MSA from CH_3S oxidation measured by nitrate and iodide ionisation. Given concentrations of H_2SO_4 and MSA are based on absolute H_2SO_4 calibrations with an uncertainty of ~20% for both ionisation schemes. Experiments on the OH + CH_3SH reaction were carried out in the free-jet flow system with a reaction time of 7.9 s, a relative humidity of 10%, $[CH_3SH] = 6.6 \times 10^{11}$ molecules cm^{-3} and OH radical production from isopropyl nitrite (IPN) photolysis with $[IPN] = (2.0 - 20) \times 10^{11}$ molecules cm^{-3}, see also Fig. 2a. Source data are provided as a Source Data file.

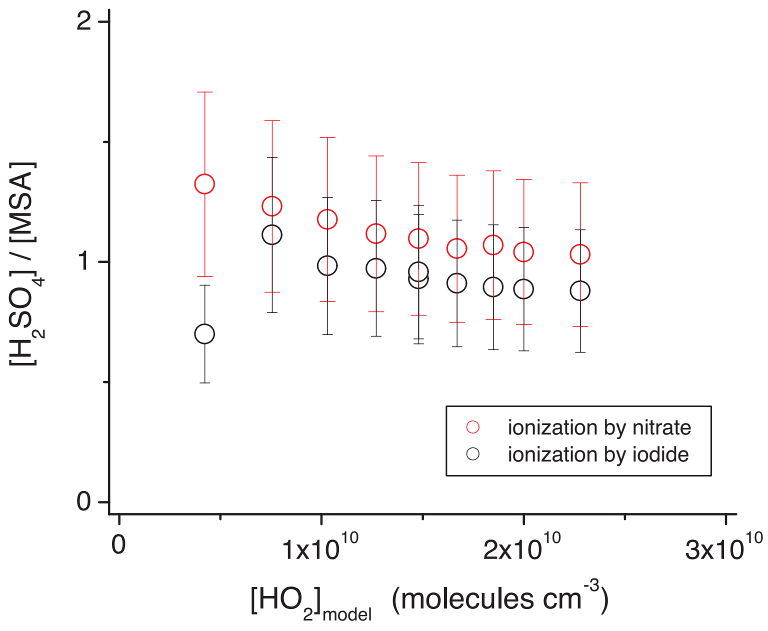

Supplementary Fig. 3: Ratio H_2SO_4 / MSA as a function of calculated HO_2 concentration. The data of H_2SO_4 and MSA were taken from the experiments shown in Fig. 2a and Supplementary Fig. 2. Error bars represent an uncertainty of 28% (Gauß´s propagation of uncertainties) based on the uncertainty of 20% in the absolute H_2SO_4 calibration. Calculated HO_2 concentrations from an extended model (Supplementary Table 4) span a range of (4.2 - 23) × 10^9 molecules cm^{-3}. An increase in HO_2 by a factor of ~5.5 should result in a strong decrease of the ratio H_2SO_4 / MSA according to the current mechanistic understanding of MSA formation, see the competing steps 17 vs. 20 in Fig. 1. The experimental finding is in contradiction to that. Source data are provided as a Source Data file.

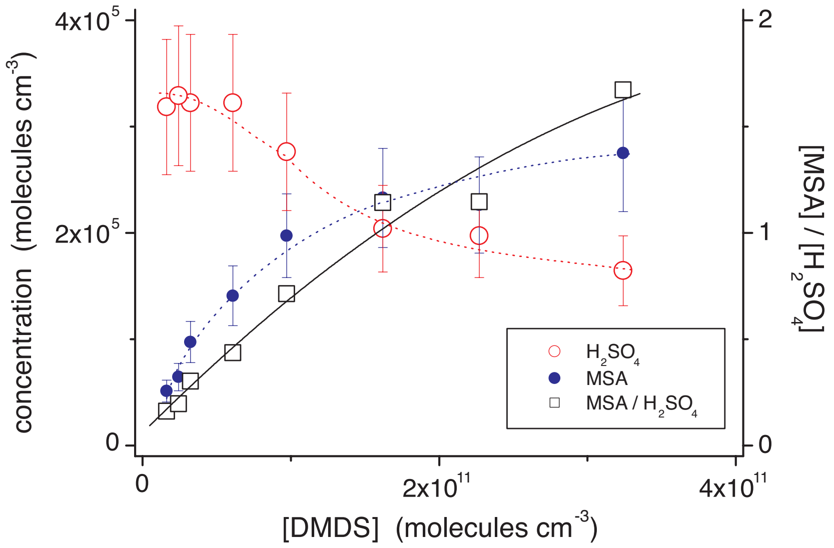

Supplementary Fig. 4: Concentrations of H_2SO_4 and MSA and their ratio as a function of DMDS concentration. Experiments on OH + DMDS were carried out in the laminar flow tube (LFT) with a reaction time of 32 s, a relative humidity of 10%, using TME ozonolysis for OH production and nitrate ionisation for product detection. Reactant concentrations were [DMDS] = (2.4 - 32.4) × 10^{10}, $[O_3]$ = 5.7 × 10^{11} and [TME] = 5.0 × 10^9 molecules cm^{-3}. The error bars depict the uncertainty of ~20% based on the uncertainty in the calibration factor. The amount of reacted DMDS was slightly variable in the course of the measurement series rising from 7.5 × 10^7 to 8.2 × 10^7 molecules cm^{-3} with increasing DMDS concentration in the reaction gas. For a DMDS concentration of 6.1 × 10^{10} molecules cm^{-3}, used in almost all experiments in the LFT, MSA and H_2SO_4 were measured with a ratio of about 0.5, see also Supplementary Figs. 6 and 8. Considering the competition between pathways 17 vs. 19, almost exclusive H_2SO_4 formation can be expected for DMDS concentrations < 10^{10} molecules cm^{-3} leading to a ~50% higher H_2SO_4 concentration than observed for [DMDS] = 6.1 × 10^{10} molecules cm^{-3} assuming constant CH_3SO_3 formation. Source data are provided as a Source Data file.

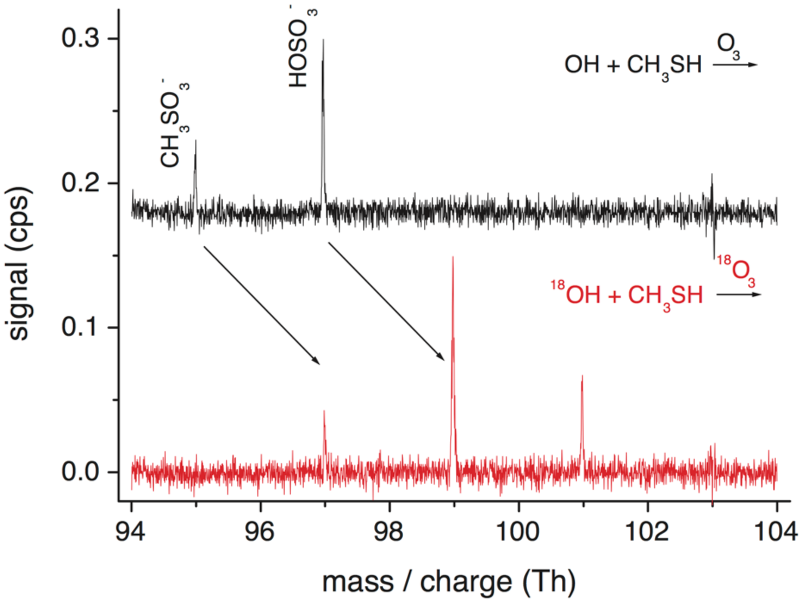

Supplementary Fig. 5: Product spectra measured from the OH + CH_3SH reaction in the presence of "normal" ozone or heavy ozone ($^{18}O_3$) using nitrate ionisation. The experiments were carried out in the laminar flow tube (LFT) with a reaction time of 32 s, a relative humidity of 10%, [CH_3SH] = 2.0 × 10^{11}, [TME] = 5.0 × 10^9 and [O_3] = 1.3 × 10^{12} molecules cm^{-3}. Heavy ozone $^{18}O_3$ was produced in the ozone generator starting from $^{18}O_2$. The $^{18}O_3$ concentration was assumed to be similar to that of the "normal" ozone. $^{18}O_2$ did not influence the further oxidation of CH_3S in the case of $^{18}O_3$ experiments due to the high oxygen ratio $^{16}O_2$ / $^{18}O_2$ = 630 in the flow tube. Heavy ozone formed heavy hydroxyl radicals ^{18}OH from TME ozonolysis, which initiated the CH_3SH reaction. The upper part shows the deprotonation signals of MSA (CH_3SO_3H) and H_2SO_4 at 94.89 and 96.98 Th, respectively, measured in the presence of "normal" ozone. Both signals are shifted by two mass units in the presence of $^{18}O_3$ (lower part) indicating the insertion of one ^{18}O atom. This behaviour is consistent with the reaction sequence 1/-1, 2 and 9 (Fig. 1). The origin of the signal observed at nominal 101 Th is not clear. It could be speculated that a smaller fraction of H_2SO_4 also contained two ^{18}O atoms. However, a mechanistic explanation for that is speculative at the moment. The spectrum measured in the presence of $^{18}O_3$ did not show the expected H_2SO_4 signal with three ^{18}O atoms at nominal 103 Th making H_2SO_4 formation via the reaction sequence 3, 6b and 9 (Fig. 1) negligible. Source data are provided as a Source Data file.

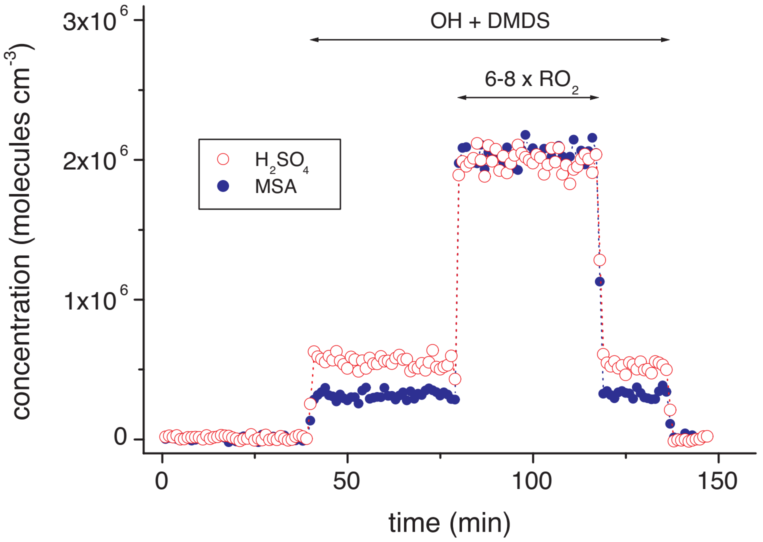

Supplementary Fig. 6: H_2SO_4 and MSA formation from OH + DMDS depending on RO_2 radical concentrations. The experiment was conducted in the LFT at r.h. = 10% using TME ozonolysis for OH production and nitrate ionisation for product detection. Reactant concentrations were [DMDS] = 6.1×10^{10}, [TME] = 1.0×10^{10} and [O_3] = 5.7×10^{11} molecules cm^{-3}. The TME concentration was increased at 1.0×10^{11} molecules cm^{-3} and CH_4 was added, [CH_4] = 2.0×10^{16} molecules cm^{-3}, during the experiment time of 79 - 117 min. The calculated end concentration of $CH_3C(O)CH_2O_2$ radicals from an extended model (Supplementary Table 4) increased from 1.2×10^8 to 1.0×10^9 and for CH_3O_2 radicals from 1.6×10^8 to 9.4×10^8 molecules cm^{-3}. Reacted DMDS was kept unchanged at 1.5×10^8 molecules cm^{-3}. The H_2SO_4 production increased by a factor of 3.8 for conditions of enhanced RO_2 radical level while the MSA production increased by a factor of 6.9. Source data are provided as a Source Data file.

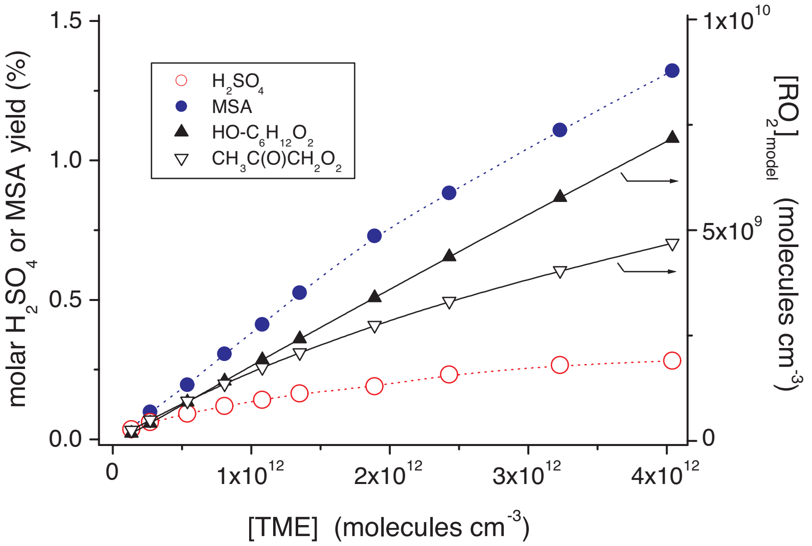

Supplementary Fig. 7: H_2SO_4 and MSA formation from OH + CH_3SH depending on RO_2 radical concentrations. The experiment has been done in the LFT at r.h. = 10% using TME ozonolysis for OH production and nitrate ionisation for product detection. Reactant concentrations were [CH_3SH] = 2.0 × 10^{10}, [O_3] = 1.5 × 10^{11} and [TME] = (1.35 - 40.4) × 10^{11} molecules cm^{-3}. With rising TME, more $CH_3C(O)CH_2O_2$ and OH radicals were formed, whereby OH primarily reacted with TME under the chosen conditions forming HO-$C_6H_{12}O_2$ radicals. In the course of the experiment, the calculated end concentration of $CH_3C(O)CH_2O_2$ increased from 2.5 × 10^8 to 4.7 × 10^9, for HO-$C_6H_{12}O_2$ radicals from 1.8 × 10^8 to 7.2 × 10^9 and for CH_3O_2 radicals only from 4.4 × 10^7 to 6.1 × 10^7 molecules cm^{-3} (Supplementary Table 4). The molar formation yields of H_2SO_4 and MSA increased from 0.036 to 0.28% and from 0.039 to 1.3%, respectively, for reacted CH_3SH of (1.8 - 2.5) × 10^8 molecules cm^{-3}. The rise in MSA seems to be tightly connected with the rise in the HO-$C_6H_{12}O_2$ radical concentration pointing to a preferred route of MSA formation induced by HO-$C_6H_{12}O_2$ radicals. Source data are provided as a Source Data file.

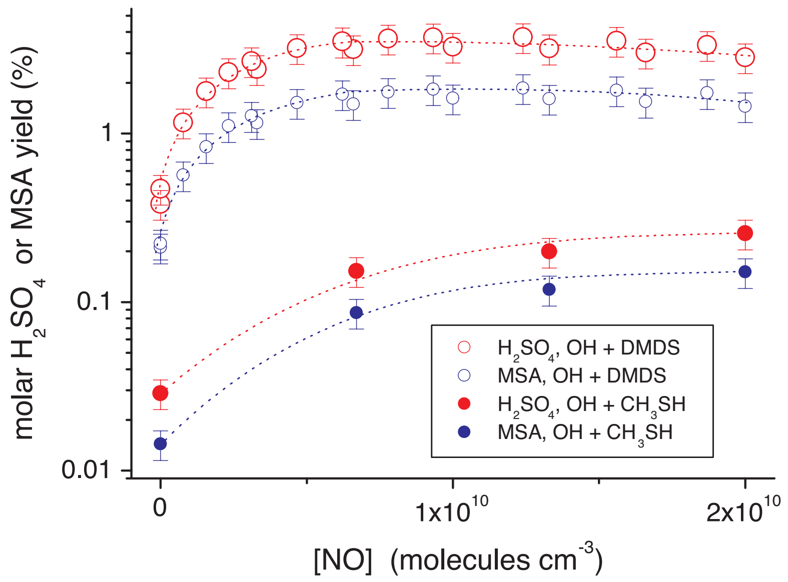

Supplementary Fig. 8: Formation of H_2SO_4 and MSA as function of NO additions using ionisation by nitrate. Experiments were conducted in the laminar flow tube (LFT), t = 32 s, at r.h. = 10% using TME ozonolysis for OH production, see also Fig. 4c of the main body. Reactant concentrations were $[O_3] = 5.7 \times 10^{11}$, [TME] = 5.0 or 15×10^9 and $[CH_3SH] = 2.0 \times 10^{11}$ or $[DMDS] = 6.1 \times 10^{10}$ molecules cm^{-3}. Error bars represent the uncertainty of ~20% in the absolute calibration. Source data are provided as a Source Data file.

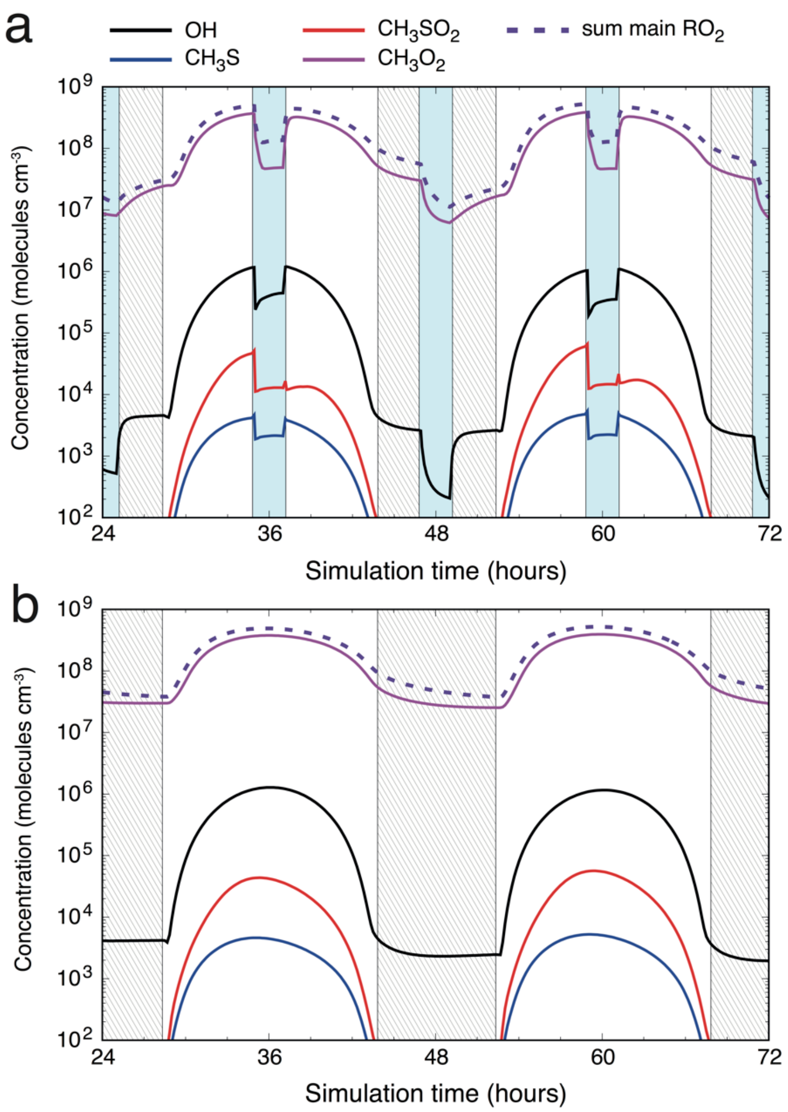

Supplementary Fig. 9: Modelling results of OH, CH_3S, CH_3SO_2 and main RO_2 radicals. Main RO_2 radicals comprise the sum of CH_3O_2, $C_2H_5O_2$, $C_3H_7O_2$, $CH_3C(O)O_2$, $C_2H_5C(O)O_2$, $CH_3CH(OH)CH_2O_2$, $CH_3CH(O_2)CH_2OH$, $CH_3SCH_2O_2$ and $HOCH_2CH_2O_2$.The grey shaded bars represent the night time, whereas the light blue bar represents the cloud occurrence when clouds are considered. **a.** "Cloud" scenario **b.** "no Cloud" scenario. Source data are provided as a Source Data file.

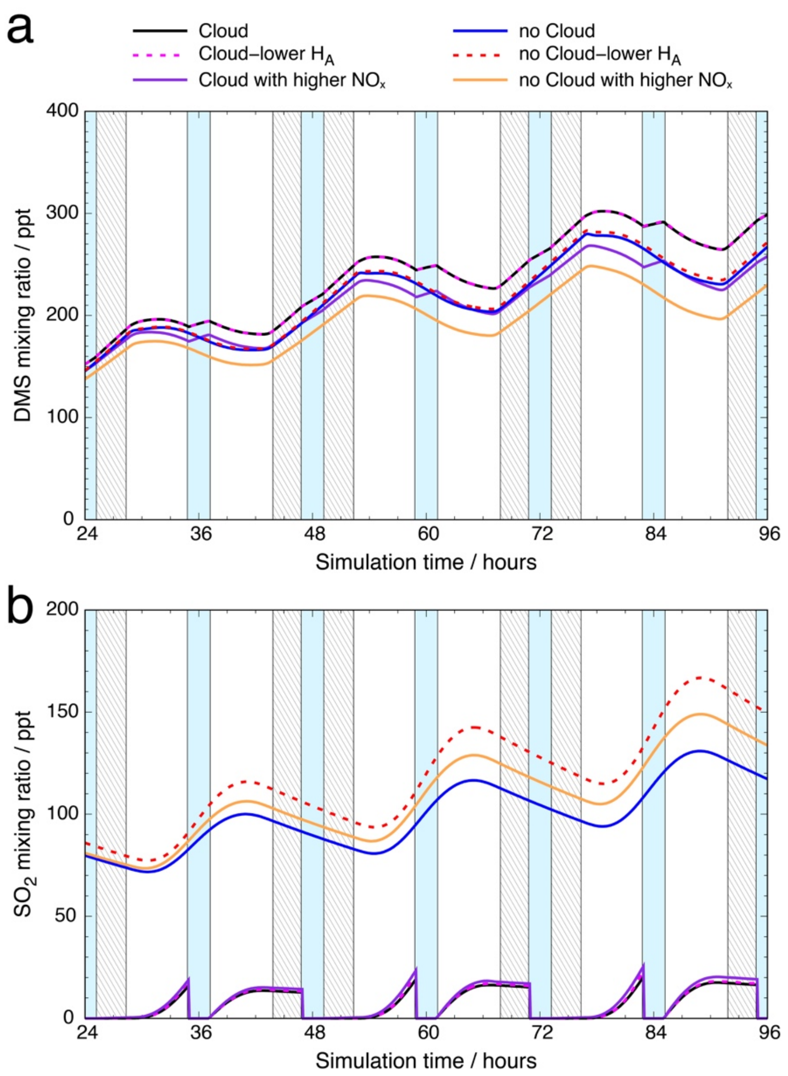

Supplementary Fig. 10: Modelling results of DMS and SO_2 mixing ratios. a. Modelled DMS mixing ratio for the different simulations from the second to the fourth model day. **b.** Modelled SO_2 mixing ratio for the different simulations from the second to the fourth model day. The grey shaded bars represent the night time, whereas the light blue bar represents the cloud occurrence when clouds are considered. Source data are provided as a Source Data file.

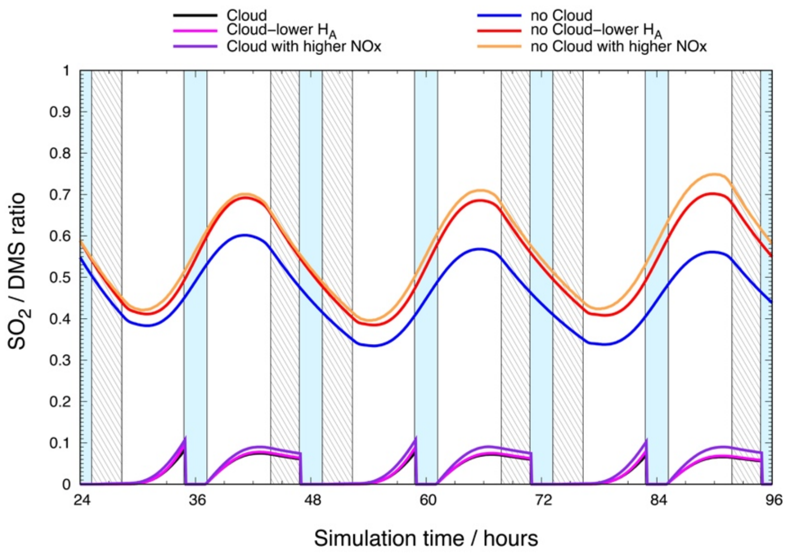

Supplementary Fig. 11: Modelling results of the SO_2 / DMS ratio. Modelled SO_2 / DMS concentration ratio for the different simulation scenarios from the second to the fourth model day. The grey shaded bars represent the night time, whereas the light blue bar represents the cloud occurrence when clouds are considered. Source data are provided as a Source Data file.

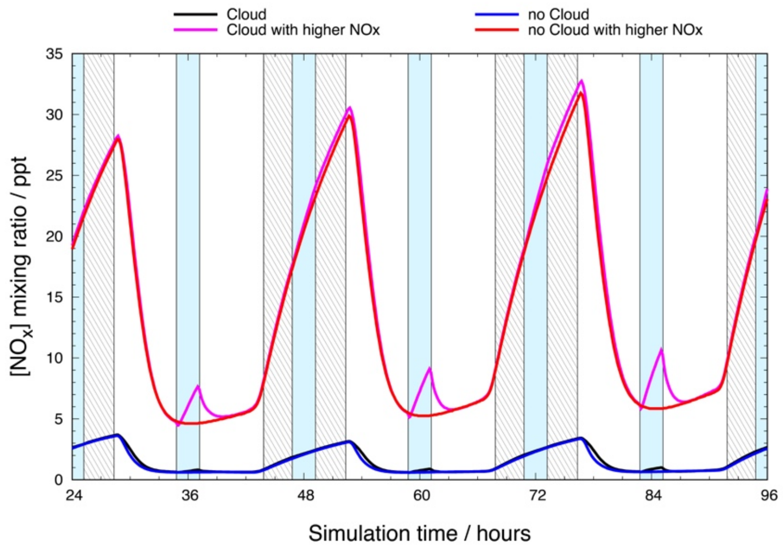

Supplementary Fig. 12: Modelling results for NO_x. Modelled NO_x mixing ratios for the different simulation cases from the second to the fourth model day, except the ones with lower H_A values, because of similar concentrations and time profiles of the NO_x mixing ratios in the simulations with lower H_A compared to the H_A Default simulations. The grey shaded bars represent the night-time, whereas the light blue bar represents the cloud occurrence when clouds are considered. Source data are provided as a Source Data file.

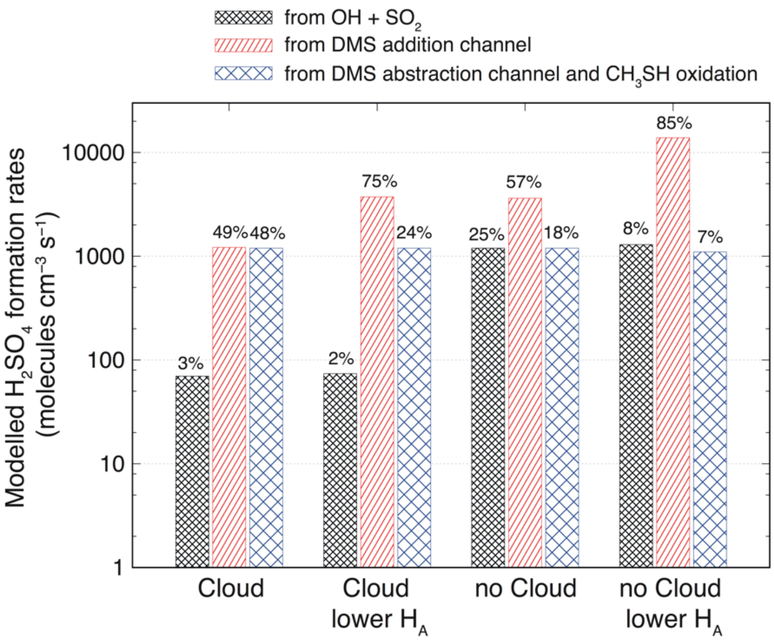

Supplementary Fig. 13: Average daytime formation rates of H_2SO_4 from OH + SO_2 and from the direct pathways of DMS and CH_3SH oxidation. The bars illustrate the modelled daytime formation rates of H_2SO_4 from OH + SO_2 (black bars) and DMS- and CH_3SH-related reaction pathways (red and blues bars) considered in the MCM/CAPRAM mechanism for the four different performed simulation scenarios. The modelled daytime formation rates were averaged from the second, third and fourth simulation day. Source data are provided as a Source Data file.

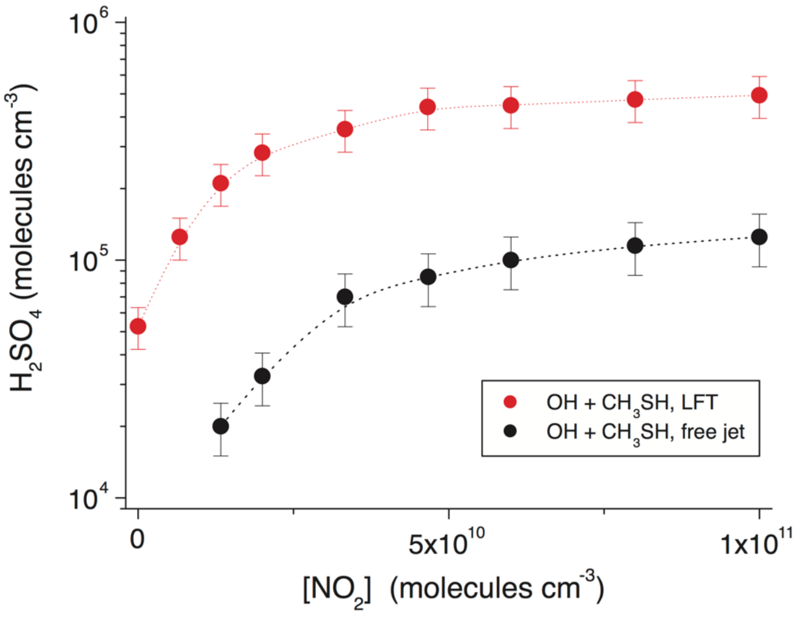

Supplementary Fig. 14: H_2SO_4 concentrations measured as a function of NO_2 in both flow systems. Experiments on OH + CH_3SH were carried out with a relative humidity of 10%, using TME ozonolysis for OH production and nitrate ionization for product detection, see also Fig. 4d. The error bars depict the uncertainty of ~20% based on the uncertainty in the calibration factor. Reactant concentrations were $[CH_3SH] = 2.0 \times 10^{10}$, $[O_3] = 5.7 \times 10^{11}$ and [TME] = 1.5 or 5.0×10^{10} molecules cm^{-3}. For measurements with $[H_2SO_4] > 5 \times 10^4$ molecules cm^{-3}, the ratio in measured H_2SO_4 concentrations in both flow systems was 4.5 ± 0.6. Source data are provided as a Source Data file.

Supplementary References

1 Wollesen de Jonge, R. *et al.* Secondary aerosol formation from dimethyl sulfide – improved mechanistic understanding based on smog chamber experiments and modelling. *Atmos. Chem. Phys.* **21**, 9955-9976, doi:10.5194/acp-21-9955-2021 (2021).

2 Witter, M., Berndt, T., Böge, O., Stratmann, F. & Heintzenberg, J. Gas-phase ozonolysis: Rate coefficients for a series of terpenes and rate coefficients and OH yields for 2-methyl-2-butene and 2,3-dimethyl-2-butene. *Int. J. Chem. Kinet.* **34**, 394-403, doi:10.1002/kin.10063 (2002).

3 Atkinson, R. *et al.* Evaluated Kinetic and Photochemical Data for Atmospheric Chemistry: Supplement IV. IUPAC Subcommittee on Gas Kinetic Data Evaluation for Atmospheric Chemistry. *J. Phys. Chem. Ref. Data* **21**, 1125-1568, doi:10.1063/1.555918 (1992).

4 Atkinson, R. *et al.* Evaluated kinetic and photochemical data for atmospheric chemistry: Volume I - gas phase reactions of O_x, HO_x, NO_x and SO_x species. *Atmos. Chem. Phys.* **4**, 1461-1738, doi:10.5194/acp-4-1461-2004 (2004).

5 Atkinson, R. *et al.* Evaluated Kinetic, Photochemical and Heterogeneous Data for Atmospheric Chemistry: Supplement V. IUPAC Subcommittee on Gas Kinetic Data Evaluation for Atmospheric Chemistry. *J. Phys. Chem. Ref. Data* **26**, 521-1011, doi:10.1063/1.556011 (1997).

6 Peltola, J., Seal, P., Vuorio, N., Heinonen, P. & Eskola, A. Solving the discrepancy between the direct and relative-rate determinations of unimolecular reaction kinetics of dimethyl-substituted Criegee intermediate $(CH_3)_2COO$ using a new photolytic precursor. *Phys. Chem. Chem. Phys.* **24**, 5211-5219, doi:10.1039/d1cp02270a (2022).

7 Cox, R. A. *et al.* Evaluated kinetic and photochemical data for atmospheric chemistry: Volume VII – Criegee intermediates. *Atmos. Chem. Phys.* **20**, 13497-13519, doi:10.5194/acp-20-13497-2020 (2020).

8 Berndt, T. *et al.* SO_2 formation and peroxy radical isomerization in the atmospheric reaction of OH radicals with dimethyl disulfide. *Chem. Commun. (Camb)* **56**, 13634-13637, doi:10.1039/d0cc05783e (2020).

9 Wennberg, P. O. *et al.* Gas-Phase Reactions of Isoprene and Its Major Oxidation Products. *Chemical Reviews* **118**, 3337-3390, doi:10.1021/acs.chemrev.7b00439 (2018).

10 Atkinson, R. *et al.* Evaluated Kinetic, Photochemical and Heterogeneous Data for Atmospheric Chemistry: Supplement V. IUPAC Subcommittee on Gas Kinetic Data Evaluation for Atmospheric Chemistry. *J. Phys. Chem. Ref. Data* **26**, 521-1011, doi:10.1063/1.556011 (1997).

11 Bridier, I., Veyret, B., Lesclaux, R. & Jenkin, M. E. Flash photolysis study of the UV spectrum and kinetics of reactions of the acetonylperoxy radical. *J. Chem. Soc. Faraday Trans.* **89**, doi:10.1039/ft9938902993 (1993).

12 Jenkin, M. E. & Hayman, G. D. Kinetics of reactions of primary, secondary and tertiary β-hydroxy peroxyl radicals. *J. Chem. Soc., Faraday Trans.* **91**, 1911-1922, doi:10.1039/ft9959101911 (1995).

13 Domine, F., Ravishankara, A. R. & Howard, C. J. Kinetics and mechanisms of the reactions of methylthio, methylsulfinyl, and methyldithio radicals with ozone at 300 K and low pressures. *J. Phys. Chem.* **96**, 2171-2178, doi:10.1021/j100184a027 (2002).

14 Turnipseed, A. A., Barone, S. B. & Ravishankara, A. R. Observation of methylthiyl radical addition to oxygen in the gas phase. *J. Phys. Chem.* **96**, 7502-7505, doi:10.1021/j100198a006 (1992).

15 Barone, S. B., Turnipseed, A. A. & Ravishankara, A. R. Role of adducts in the atmospheric oxidation of dimethyl sulfide. *Faraday Discuss.* **100**, doi:10.1039/fd9950000039 (1995).

16 Khan, M. A. H. *et al.* Impacts of Hydroperoxymethyl Thioformate on the Global Marine Sulfur Budget. *ACS Earth Space Chem.* **5**, 2577-2586, doi:10.1021/acsearthspacechem.1c00218 (2021).
17 MCM v3.2 (references: Jenkin, M. E., Saunders, S. M. & Pilling, M. J. The tropospheric degradation of volatile organic compounds: a protocol for mechanism development. *Atmos. Environ.* **31**, 81-104, doi:10.1016/S1352-2310(96)00105-7 (1997); Saunders, S. M., Jenkin, M. E., Derwent, R. G. & Pilling, M. J. *Atmos. Chem. Phys.* **3**, 161-180, doi:10.5194/acp-3-161-2003 (2003)), via website: http://mcm.leeds.ac.uk/MCM.
18 Wu, R., Wang, S. & Wang, L. New mechanism for the atmospheric oxidation of dimethyl sulfide. The importance of intramolecular hydrogen shift in a CH_3SCH_2OO radical. *J. Phys. Chem. A* **119**, 112-117, doi:10.1021/jp511616j (2015).
19 Jenkin, M. E., Valorso, R., Aumont, B., Rickard, A. R. & Wallington, T. J. Estimation of rate coefficients and branching ratios for gas-phase reactions of OH with aliphatic organic compounds for use in automated mechanism construction. *Atmos. Chem. Phys.* **18**, 9297-9328, doi:10.5194/acp-18-9297-2018 (2018).
20 Aranda, A., Díaz de Mera, Y., Rodríguez, D., Salgado, S. & Martínez, E. Kinetic and products of the BrO + CH_3SH reaction: temperature and pressure dependence. *Chem. Phys. Lett.* **357**, 471-476, doi:10.1016/s0009-2614(02)00561-4 (2002).
21 Hoffmann, E. H. *et al.* An advanced modeling study on the impacts and atmospheric implications of multiphase dimethyl sulfide chemistry. *Proc. Natl. Acad. Sci. U S A* **113**, 11776-11781, doi:10.1073/pnas.1606320113 (2016).
