# Supplementary material for: Direct sulfuric acid formation from the gas-phase oxidation of reduced-sulfur compounds
Source: Nat Commun. 2023 Aug 10;14:4849. doi: 10.1038/s41467-023-40586-2 (PMC10415363; doi:10.1038/s41467-023-40586-2)
Supplement: Supplementary file 3 — Description of Additional Supplementary Files [file 41467_2023_40586_MOESM3_ESM.pdf]

Description of Additional Supplementary Files:

Supplementary Dataset 1: The measurement data collected from the literature and used in this work are provided in the attached Supplementary Dataset 1.
